# Telomere length-dependent transcription and epigenetic modifications in promoters remote from telomere ends

**DOI:** 10.1371/journal.pgen.1007782

**Published:** 2018-11-15

**Authors:** Ananda Kishore Mukherjee, Shalu Sharma, Suman Sengupta, Dhurjhoti Saha, Pankaj Kumar, Tabish Hussain, Vivek Srivastava, Sumitabho Deb Roy, Jerry W. Shay, Shantanu Chowdhury

**Affiliations:** 1 Genomics and Molecular Medicine Unit, CSIR-Institute of Genomics and Integrative Biology, New Delhi, India; 2 Academy of Scientific and Innovative Research, CSIR-Institute of Genomics and Integrative Biology, New Delhi, India; 3 G.N.R. Knowledge Centre for Genome Informatics, CSIR-Institute of Genomics and Integrative Biology, New Delhi, India; 4 Department of Cell Biology, University of Texas Southwestern Medical Center, Dallas, Texas, United States of America; Chinese Academy of Sciences, CHINA

## Abstract

Telomere-binding proteins constituting the shelterin complex have been studied primarily for telomeric functions. However, mounting evidence shows non-telomeric binding and gene regulation by shelterin factors. This raises a key question—do telomeres impact binding of shelterin proteins at distal non-telomeric sites? Here we show that binding of the telomere-repeat-binding-factor-2 (TRF2) at promoters ~60 Mb from telomeres depends on telomere length in human cells. Promoter TRF2 occupancy was depleted in cells with elongated telomeres resulting in altered TRF2-mediated transcription of distal genes. In addition, histone modifications—activation (H3K4me1 and H3K4me3) as well as silencing marks (H3K27me3)—at distal promoters were telomere length-dependent. These demonstrate that transcription, and the epigenetic state, of telomere-distal promoters can be influenced by telomere length. Molecular links between telomeres and the extra-telomeric genome, emerging from findings here, might have important implications in telomere-related physiology, particularly ageing and cancer.

## Author summary

Telomeres (special DNA-protein assemblies that protect chromosome ends) affect ageing and diseases such as cancer. Although this has been recognized for many years, biological processes that connect telomeres to ageing, cancer and other cellular functions remain to be fully understood. Certain proteins, believed to be only telomere-associated, engage DNA outside telomeres. This raises an interesting question. Does telomere length influence how telomere-binding proteins associate with DNA at regions distal from telomeres. If so, how does this impact function? Motivated by these questions, in the present studies we tested if extra-telomeric binding of the well-known telomere-repeat-binding-actor-2 (TRF2) depends on telomere length. Our results show that the level of DNA-bound TRF2 at telomere-distal sites changes as telomeres shorten or elongate. Consequently, TRF2-mediated gene regulation affects many genes. Notably, histone modifications that dictate chromatin compaction and access to regulatory factors, at sites distant from telomere ends also depended on telomere length. Together, this links the state of telomeres to gene regulation and epigenetics directly in ways not previously appreciated that might impact a more complete understanding of molecular processes underlying ageing and cancer.

## Introduction

The shelterin complex comprises a group of proteins that confer stability to telomeres[1–6]. Components of shelterin and other telomeric proteins, for example, RAP1, TRF1 and TRF2, and POT1 have been extensively studied in telomere homeostasis[7–10], DNA replication[11–15], repair and genomic stability[16–21]. Previous findings implicate shelterin members in non-telomeric functions related to their presence at distal regions outside telomeres[22–29]. Telomere-independent RAP1 was reported to influence global gene expression through associations with extra-telomeric DNA[23] and also by directly associating with the IkappaB kinase complex thereby inducing NFKappaB-dependent gene expression[24,30]. In addition to RAP1 engaging non-telomeric DNA, another shelterin component TRF2[31] was detected to bind DNA in about 200 sites genome wide[25,26]. Furthermore, TRF2-mediated transcription regulation was reported for *PDGFR-beta*[27] and recently promoter-binding of TRF2 was reported to repress the cyclin-dependent kinase *p21* (*CDKN1A/CIP1/WAF1*) through TRF2-dependent recruitment of the REST-LSD1 repressor complex[28].

Together these implicate functions of shelterin proteins beyond telomeres. They also, contextually, raise the question about whether and how shelterin components might link telomeres to non-telomeric cellular functions. Herein we ask this question specifically focusing on TRF2. We examined telomeric and non-telomeric TRF2 occupancy in human cells with short vis-à-vis long telomeres. Proportional increase in TRF2 occupancy at telomeres was evident in cells with enhanced telomere length consistent with a previous report[32]. Importantly, TRF2 occupancy was significantly depleted at many non-telomeric promoter sites across the genome in cells with elongated telomeres. The promoters were located at varying distances from telomere ends ranging from ~3 to 60 Mb. In addition, reduced promoter TRF2 occupancy in cells with elongated versus short telomeres resulted in altered gene expression. We noted that epigenetic histone modifications at the promoters, though remote from telomeres, were dependent on telomere length; that is, increase (or decrease) in activation marks H3K4me1/H3K4me3, (along with reduction (or increase) in H3K27me3) in respective promoters consistent with activation/repression of the gene was observed in all cases. Functionally, this shows TRF2-mediated transcription dependent on TRF2 promoter occupancy in cells with elongated/short telomeres. Taken together, these findings suggest that epigenetic changes and transcription at promoters remote from telomeres are telomere-length dependent.

## Results

### TRF2 binding at many promoters affects gene transcription

**Fig 1:**
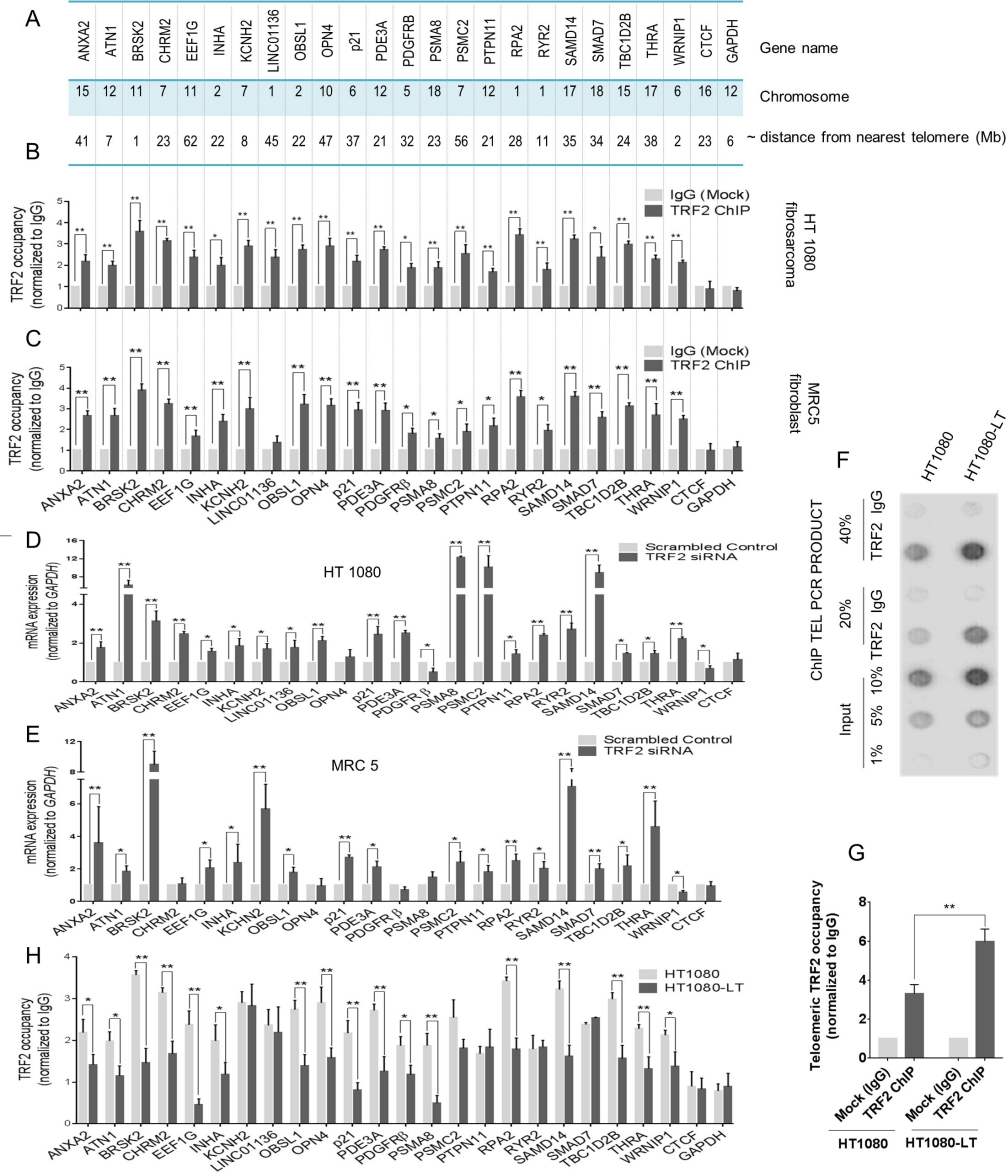
Significantly altered TRF2 occupancy at non-telomeric sites in cells with short versus long telomeres. **A. Distance of selected promoters from nearest telomere end.** Gene promoters with TRF2 binding sites (within 1.5 kb of transcription start sites (TSS)) selected from replicate ChIP-seq studies (raw data publicly available-SRA 304653) and published reports of extra-telomeric occupancy of TRF2. **B-C. Promoter occupancy of TRF2.** TRF2 occupancy at gene promoters was checked by ChIP-qPCR in HT1080 (B) and MRC5 cells (C). *CTCF* and *GAPDH* promoters with no TRF2 binding within +/- 5 kb of TSS were used as negative controls. Error bars indicate ± SD from three independent experiments; significance was tested by paired T-test -* <0.05; **<0.01. **D-E. TRF2 silencing transcriptionally affects gene expression.** Effect of TRF2 silencing on gene promoters was tested in HT1080 (D) and MRC5 cells (E). *CTCF w*as used as a negative control gene; normalized with respect to GAPDH expression. Error bars indicate ± SD from three independent experiments; significance was tested by paired T-test -* <0.05; **<0.01. **F-G. HT1080-LT cells with long telomeres have more telomeric TRF2 occupancy in comparison to HT1080 cells.** ChIP with TRF2 antibody (or isotypic control) was followed by PCR using telomere-specific primer (TEL-PCR) in HT1080 and HT1080-LT cells (F). Input samples and TEL-PCR products were blotted on membrane and hybridized with telomere-specific probes. Quantification of three independent dot blot assays (G). Error bars indicate ± SD from three independent experiments; significance was tested by paired T-test -* <0.05; **<0.01. **H. Significantly reduced TRF2 occupancy at gene promoters in cells with long telomeres.** TRF2 occupancy at many promoters was lower in HT1080-LT cells compared to HT1080 cells. *CTCF* and *GAPDH* promoters with no TRF2 binding within +/- 5 kb of TSS were used as negative controls. Error bars indicate ± SD from three independent experiments; significance was tested by paired T-test -* <0.05; **<0.01.

We sought to test TRF2 occupancy at sites distal to telomeres in cells with short vis-à-vis long telomeres. For this 8 gene promoters with extra-telomeric TRF2 binding reported by us and others were taken[26–28,33].In addition, another 15 gene promoters with putative TRF2 binding sites were randomly selected from TRF2 ChIP-seq in HT1080 cells such that the genes were located at varying distances from telomeres (~2 to 60 Mb from nearest telomere) and had TRF2 occupancy within 1.5 Kb of transcription start sites (TSS) (Fig 1A and S1 Table). Significant TRF2 occupancy was first confirmed at all the 23 promoters by ChIP-qPCR in fibrosarcoma HT1080 and normal human fibroblast MRC5 cells, with the exception of *LINC01136* promoter, which was not enriched for TRF2 occupancy in MRC5 cells (Fig 1B and 1C). No TRF2 occupancy was found within 7 negative control regions *(CTCF*, *GAPDH*, *b-actin*, *Synapsin* and *STAT2* promoters and 3’UTRs of *p21* and *SAMD14*) in HT1080 and MRC5 cells (Fig 1B and 1C and S1A Fig).

TRF2 silencing by siRNA resulted in significantly altered expression of most target genes in both the cell types. Expression of all the 23 target genes changed in HT1080 cells (Fig 1D and S1B Fig); 20 genes were up-regulated and two genes *PDGFR β* and *WRNIP1* were down-regulated (*OPN4* did not change significantly; *PDGFR β* decrease on TRF2 silencing in cancer cells was also noted earlier[27]). In MRC5 cells, out of 22 target genes (due to lack of promoter TRF2 occupancy in MRC5 cells *LINC01136* was excluded) expression of 18 genes altered significantly (Fig 1E and S1B Fig; differential expression of *CHRM2*, *OPN4*, *PDGFR β* and *PSMA8* was not significant). *WRNIP1* down-regulation was consistent in both cells lines. Increase in *p21* and *RPA2* expression in both cell lines on TRF2 silencing was consistent with previous reports[26,28] and *OPN4* did not show significant change in expression in either cell line. Expression of negative control genes *CTCF*, *b-actin*, *Synapsin* and *STAT2* did not change significantly upon TRF2 silencing in both cell types (Fig 1D and 1E and S1C Fig).

### TRF2 occupancy at promoters remote from telomeres is significantly altered in cells with short versus long telomeres

We first checked whether cells with long telomeres have more telomere-bound TRF2. The isogenic line of HT1080 cells with enhanced telomeres reported earlier[32] was used (designated as cells with long telomeres HT1080-LT cells in following text). We confirmed HT1080-LT cells had elongated telomeres compared to HT1080 cells (from ~4.5 Kb in HT1080[32,34,35] cells to average telomere length of ~8–9 Kb in LT cells; S2A and S2B Fig). Expression of both *hTERT* and *hTERC* genes, and telomerase activity was enhanced in HT1080-LT cells reaffirming the reported telomere elongation phenotype (S2C Fig). HT1080-LT cells with elongated telomeres had relatively more TRF2 occupancy at telomeres compared to HT1080 cells consistent with the earlier finding[32] (Fig 1F and 1G, S2D Fig). Total cellular and chromatin-bound TRF2 was roughly similar in HT1080-LT and HT1080 cells, and TRF2 in the nucleoplasm was significantly low compared to chromatin-bound TRF2 for the same amount of protein lysate suggesting nuclear TRF2 was mostly bound to chromatin (S2E Fig). TRF2 expression in whole cell lysate was found to be similar in HT1080 and HT1080-LT cells (S2F Fig).

Binding of TRF2 at the 23 target promoter sites validated earlier (Fig 1B) was compared in HT1080 cells with short versus long telomeres. TRF2 occupancy was significantly altered in cells with long telomeres in 17 of the 23 promoters. Notably, in all the 17 cases promoter TRF2 occupancy was depleted in HT1080-LT vis-à-vis HT1080 cells (Fig 1H). TRF2 occupancy did not change detectably in case of *KCNH2*, *LINC01136*, *PSMC2*, *PTPN11*, *RYR2* and *SMAD7*; *CTCF* and *GAPDH* promoters with no TRF2 binding were used as negative controls.

**Fig 2:**
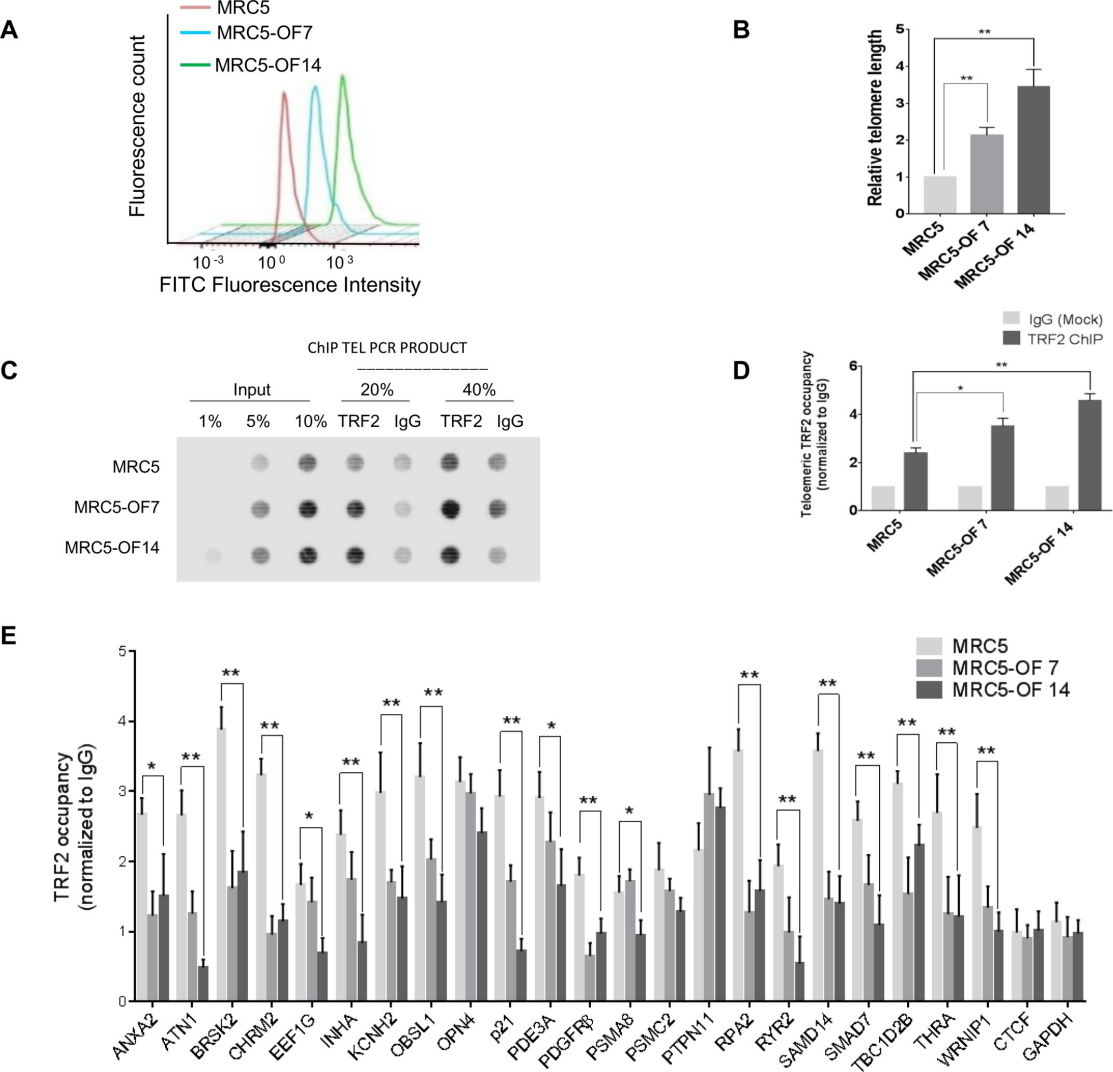
Altered promoter TRF2 occupancy in normal fibroblast cells with long or short telomeres. **A-B. Telomere elongation.** Telomere elongation in MRC5 cells was confirmed by flow-FISH using FITC-tagged telomere-specific probes following treatment with G-rich terminal repeat (GTR) oligonucleotides for either 7 (MRC5-OF7), 14 (MRC5-OF14) cycles or without treatment (MRC5) (A); also see Supplementary S2A Fig. Relative telomere length was quantified by three independent flow-FISH experiments (B). Error bars indicate ± SD from three independent experiments; significance was tested by paired T-test -* <0.05; **<0.01. **C-D. MRC5 cells with long telomeres have more telomeric TRF2**. ChIP with TRF2 antibody (or isotypic control) was followed by PCR using telomere-specific primers in MRC5 cells. Input samples and the TEL-PCR products were blotted on a membrane and hybridized with telomere-specific probes—dot blot assay showing cells with long telomeres have enriched TRF2 occupancy at telomeres in MRC5-OF7 and MRC5-OF14 cells (C). Quantification of three independent dot blot assays (D). Error bars indicate ± SD from three independent experiments; significance was tested by paired T-test -* <0.05; **<0.01. **E. Significantly lower TRF2 occupancy at promoters in cells with long telomeres.** TRF2 occupancy at multiple promoter sites was reduced in MRC5-OF7 and OF14 cells in comparison to untreated cells. *CTCF* and *GAPDH* promoters with no TRF2 binding within +/- 5 kb of TSS were used as negative controls. Error bars indicate ± SD from three independent experiments. Significance was tested by paired T-test -* <0.05; **<0.01.

To further test this we next used non-cancerous normal fibroblast MRC5 and corresponding isogenic cells with longer telomeres (made using a different mode of telomere elongation[36]). Repeated treatment of MRC5 cells with G-rich terminal oligonucleotides (GTR) over multiple passages (S3A Fig) resulted in telomere elongation as reported earlier[36]. We found ~2-3-fold elongation of telomeres in cells sequentially fed with GTR for either 7 or 14 cycles (oligo-fed OF7 or OF14 cells, respectively). Telomere elongation was from about 9 Kb in case of MRC5 cells[37] to average telomere length of either 18 Kb (MRC-OF7) or 27 Kb (MRC5-OF14) (Fig 2A and 2B and S3B Fig). Accordingly, increase in expression of *hTERT* and *hTERC* and enhanced telomerase activity was observed (S3C and S3D Fig).

Increase in telomere-bound TRF2 was found in the cells with long telomeres (OF7, OF14) relative to untreated MRC5 cells (Fig 2C and 2D, S3E Fig). Chromatin-bound TRF2 did not show significant difference in MRC5, MRC5-OF7 and MRC5-OF14 cells. In addition, similar to HT1080 cells, we noted that free TRF2 in the nucleoplasmic fraction in all the three lines was low compared to chromatin-bound TRF2 for the same amount of protein lysate suggesting nuclear TRF2 was largely bound to chromatin (S3F Fig). However, we noted ~15–20% increase in nucleoplasmic and total TRF2 in telomere-elongated MRC5 cells (S3F and S3G Fig).

Next, we asked whether non-telomeric TRF2 occupancy varied in cells with short versus long telomeres in MRC5 cells. We tested the 22 target promoters with significant TRF2 occupancy validated earlier in MRC5 cells (Fig 1C). In 19 of the 22 promoters TRF2 binding was significantly depleted in cells with long telomeres compared to untreated MRC5 cells with short telomeres (Fig 2E), similar to the observations in HT1080 cells. In case of *OPN4*, *PSMC2* and *PTPN11* altered TRF2 occupancy was not significant. Together, results obtained in HT1080 and MRC5 cells suggest that TRF2 occupancy at sites distal to telomeres was influenced by the length of telomeres.

### Transcription of the cyclin-dependent kinase *p21* (*CDKN1A/CIP1/WAF1*) is telomere length dependent

**Fig 3:**
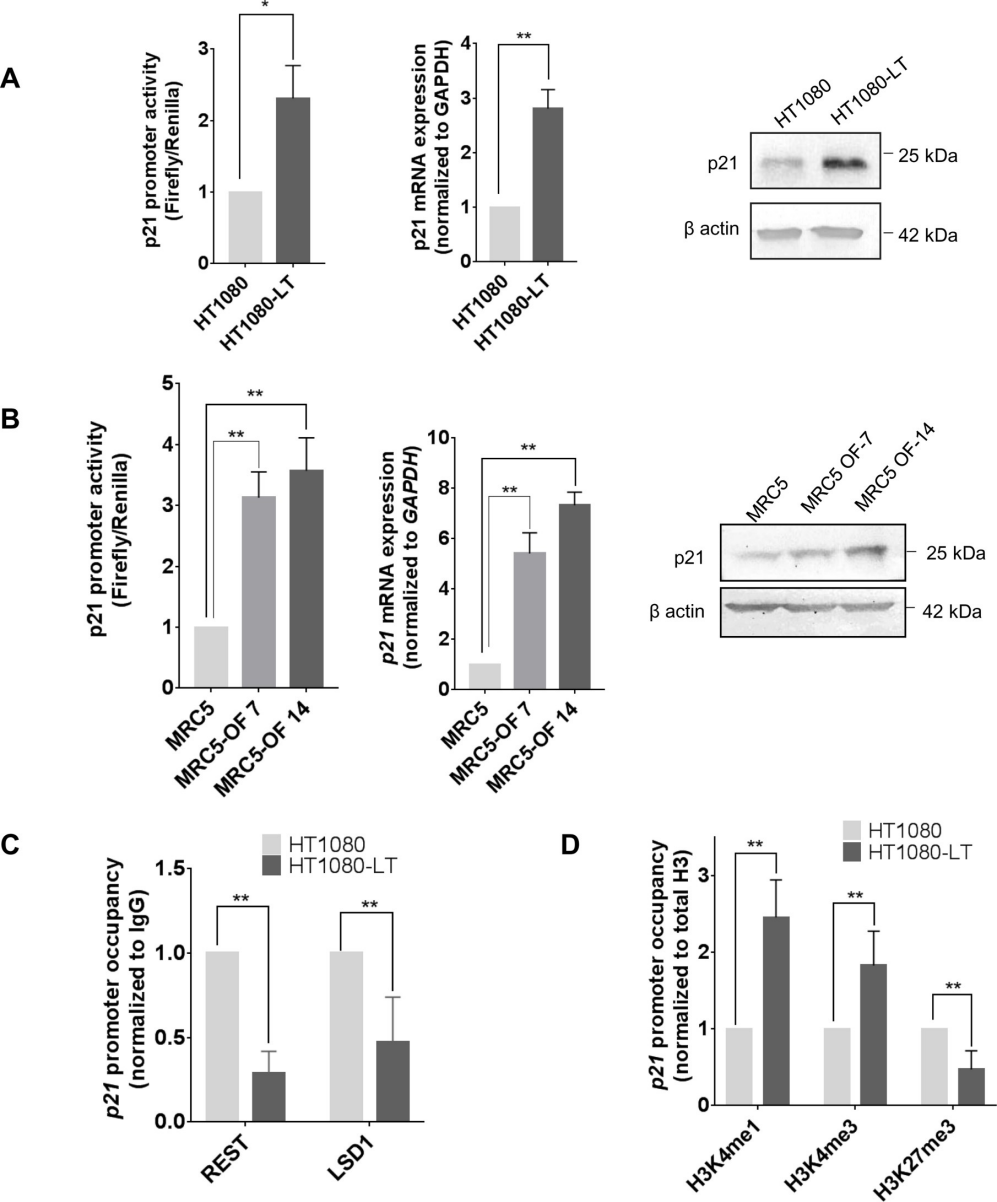
Transcription activity of *p21* is altered in cells with short vis-à-vis long telomeres. **A-B. *p21* expression in HT1080-LT and MRC5 cells**. *p21* promoter activity, mRNA and protein levels increased in HT1080-LT relative to HT1080 cells (A) and MRC5-OF7/14 compared to untreated MRC5 cells (B). Error bars indicate ± SD from three independent experiments; significance was tested by paired T-test -* <0.05; **<0.01. **C. Altered transcription of *p21* through loss of REST and LSD1 from the *p21* gene promoter.** Loss of REST and LSD1 occupancy from *p21* promoter in HT1080-LT as compared to HT1080 cells. Error bars indicate ± SD from three independent experiments; significance was tested by paired T-test -* <0.05; **<0.01. **D. Histone modifications at the *p21* promoter in cells with short/long telomeres.** HT1080-LT cells with elongated telomeres had enrichment of activating histone marks, H3K4me1 and H3K4me3, and reduction in the suppression histone mark H3K27me3. Error bars indicate ± SD from three independent experiments; significance was tested by paired T-test -* <0.05; **<0.01.

We recently reported that *p21* was transcriptionally repressed by TRF2[28]. Here we asked if altered TRF2 binding in cells with elongated telomeres affected *p21* expression. Because of reduced occupancy of TRF2 at the *p21* promoter in HT1080-LT cells (Fig 1H), we observed that *p21* promoter activity as well as mRNA and protein levels were enhanced in HT1080-LT compared to HT1080 cells with shorter telomeres (Fig 3A). Transient over expression of *hTERT* and *hTERC* in HT1080 cells did not affect TRF2 occupancy at the *p21* promoter or *p21* expression (S4A–S4C Fig). Therefore, it is unlikely that change in *p21* expression was due to any indirect effect of *hTERT* and/or *hTERC* expression used for inducing telomere elongation in HT1080 cells. Increase in *p21* promoter activity, mRNA expression, and p21 protein levels was also found in MRC5-OF7 and OF14 cells with long telomeres relative to untreated MRC5 cells (Fig 3B) consistent with reduced TRF2 occupancy at the *p21* promoter in MRC5 cells with long telomeres (Fig 2E), supporting similar observations in HT1080 cells.

Together these demonstrate that regulation of *p21* by TRF2 is dependent on whether cells have long or short telomeres showing expression of a gene remote from telomeres (~36 Mb) can be affected by telomere length.

TRF2-dependent recruitment of the RE-1 silencing transcription factor (REST) and lysine-specific demethylase 1 (LDS1) at the *p21* promoter was observed by us earlier[28]. Along with reduced TRF2 occupancy, REST and LSD1 binding at the *p21* promoter was significantly depleted in HT1080-LT when compared to HT1080 cells (Fig 3C and S5A and S5B Fig). On the other hand, REST occupancy at the *Synapsin* promoter (S1A Fig) was not different in HT1080/HT1080-LT cells (S5A Fig) indicating that TRF2-independent REST binding was not affected by telomere length. The *CTCF* promoter with no reported REST occupancy was used as a negative control (S5A Fig).

Because loss of REST and LSD1 was expected to affect the epigenetic state we analyzed the presence of activating (H3K4me1 and H3K4me3) and silencing (H3K27me3) histone modifications at the *p21* promoter. In HT1080-LT cells there was a significant increase in both activation marks H3K4me1 and H3K4me3; and reduction in suppressor mark H3K27me3 (Fig 3D). Together these argue for TRF2-dependent epigenetic changes at the *p21* promoter that are sensitive to telomere length.

### TRF2-mediated expression of many genes across the genome are influenced by telomere length

**Fig 4:**
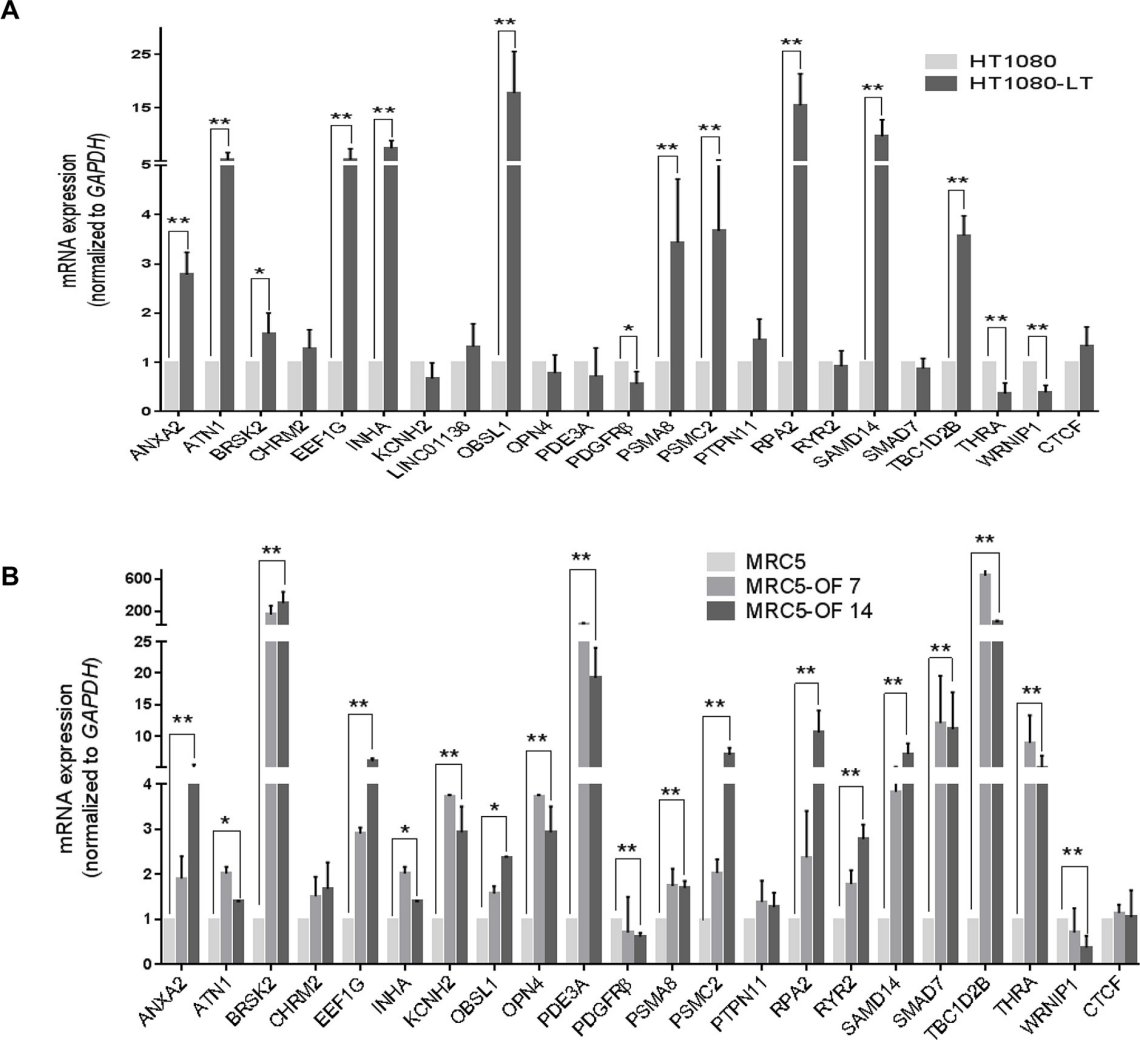
Transcription of many genes altered in cells with short compared to long telomeres. **A-B. Differential mRNA expression of genes with altered promoter occupancy of TRF2 in cells with short/long telomeres.** mRNA expression was checked by qPCR for genes in which TRF2 promoter occupancy was reduced in HT1080-LT cells compared to HT1080 cells (A), and MRC5-OF7 or OF14 cells compared to untreated MRC5 cells (B). *CTCF* was used as a negative control. Error bars indicate ± SD from three independent experiments; significance was tested by paired T-test -* <0.05; **<0.01.

We checked whether telomere length altered the expression of genes other than *p21*. Expression of 13 out of 16 genes (excluding *p21* described above), where promoter TRF2 occupancy was earlier observed to be depleted in HT1080-LT (Fig 1H), was significantly up or down regulated in HT1080-LT versus HT1080 cells (Fig 4A). This was consistent with the effect expected from TRF2 silencing in HT1080 cells (Fig 1D) and, therefore, likely from reduced promoter TRF2 occupancy in cells with long telomeres. In five promoters where TRF2 occupancy did not change in HT1080-LT/HT1080 cells expression of corresponding genes also remained unaltered (*KCNH2*, *LINC01136*, *PTPN11*, *RYR2* and *SMAD7*). Altered *PSMC2* expression was observed though TRF2 binding remained unchanged in HT1080-LT/HT1080 cells whereas *CHRM2* and *PDE3A* expression did not change despite altered TRF2 binding in short/long telomere HT1080 cells (Fig 1H). In contrast to the other target genes, *PDGFRβ* and *WRNIP1* were down-regulated on TRF2 silencing (Fig 1D) suggesting TRF2-mediated activation. This was consistent with loss of promoter TRF2 occupancy in HT1080-LT relative to HT1080 cells giving down-regulation of *PDGFRβ* and *WRNIP1* (Fig 4A).

To test whether the differential expression of 14 genes (including *p21*) in HT1080-LT cells was sensitive to *hTERT/hTERC* we overexpressed *hTERT* and/or *hTERC*. Expression of none of the 14 genes was altered by *hTERC* whereas two genes *ANXA2* (~25% up-regulation) and *TBC1D2B* (~20% down-regulation) were altered by *hTERT* (S6 Fig). In HT1080-LT cells *ANXA2* expression was up-regulated by ~3-fold (Fig 4A) suggesting a combined effect of both increased *hTERT* and loss in TRF2 promoter occupancy. *TBC1D2B* expression, on the other hand, was up-regulated >3-fold in HT1080-LT cells (Fig 4A).

Next, we overexpressed TRF2 in HT1080-LT cells to see if the effect of reduced promoter TRF2 occupancy could be reverted. Fourteen genes with both altered TRF2 occupancy and gene expression in HT1080/HT1080-LT cells were tested. TRF2 overexpression resulted in suppression of 11 of the 14 genes in HT1080-LT cells (S7A Fig). *PDGFR-beta* and *WRNIP1* were up-regulated–consistent with TRF2-mediated up-regulation noted for these genes. In case of *THRA* no significant change in expression was observed. Furthermore, we compared levels of gene expression in both HT1080/HT1080-LT cells following TRF2 overexpression. In 10 out of the 14 genes expression levels were similar (S7B Fig). Higher expression of *ANXA2* in HT1080-LT cells was likely due to increased *hTERT* (as noted independently on *hTERT* overexpression, S6 Fig).

In case of MRC5 cells, out of 21 targets (excluding *p21*) 19 genes were differentially expressed in MRC5-OF cells with elongated telomeres relative to untreated MRC5 cells (Fig 4B). Similar to HT1080 cells, for all except two (*OPN4* and *PMSC2*) of the 19 genes, TRF2 promoter occupancy was also significantly reduced in long telomeres (MRC5-OF) compared to untreated MRC5 cells (Fig 2E). *CHRM2* expression did not change though TRF2 binding was altered in short/long MRC5 cells, as was noted in HT1080 cells. *PTPN11* expression remained unchanged as expected from unaltered TRF2 promoter occupancy in MRC5-OF versus MRC5 cells, which was consistent across both the telomere elongation models.

### Epigenetic histone modifications at many promoters remote from telomeres linked to telomere length

**Fig 5:**
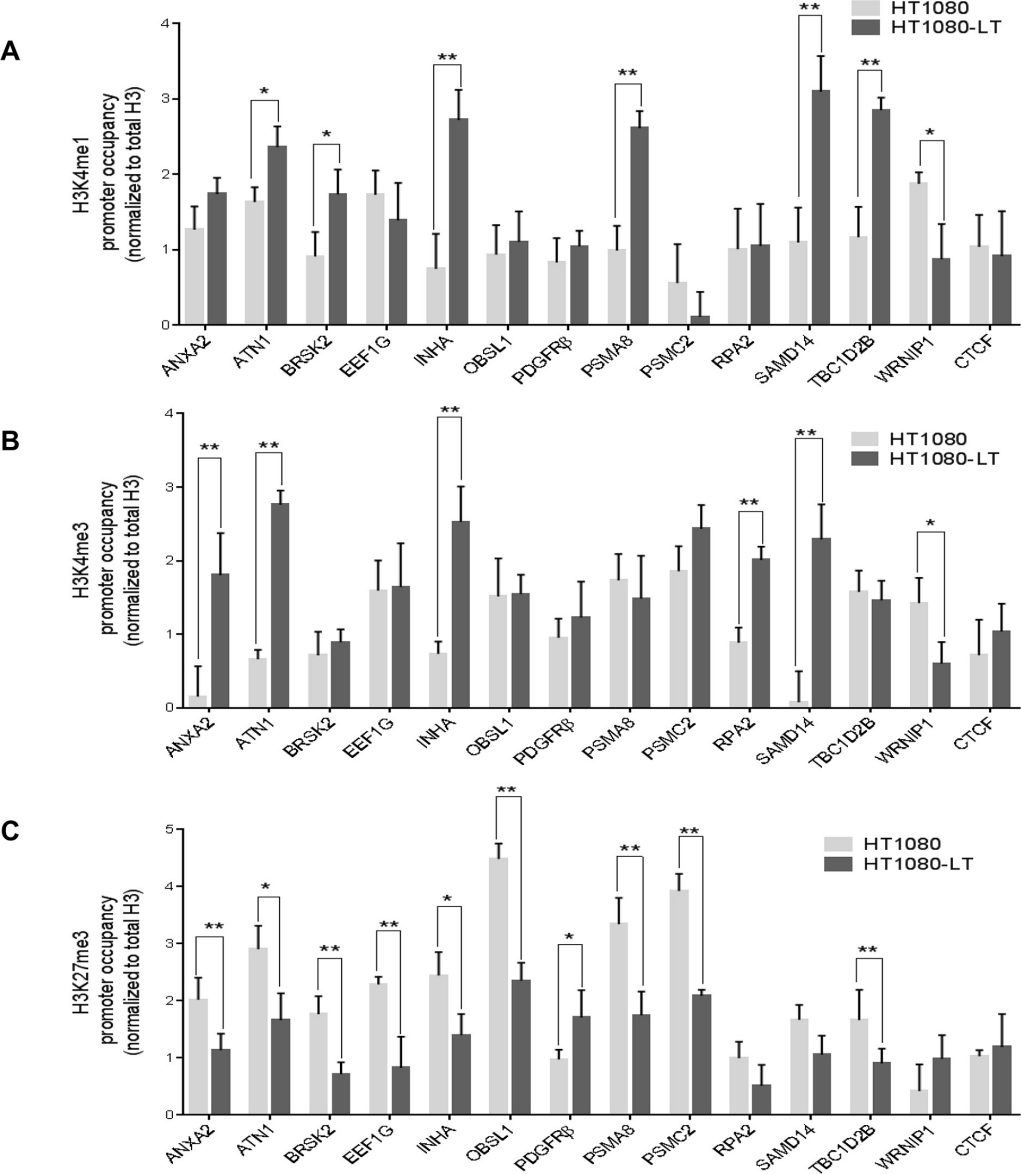
Promoter epigenetic modifications altered in cells with short versus long telomeres. **A-C.** Promoter occupancy H3K4me1 (A), H3K4me3 (B) and H3K27me3 (C) in HT1080-LT relative to HT1080 cells. Error bars indicate ± SD from three independent experiments; significance was tested by paired T-test -* <0.05; **<0.01.

Based on the histone modifications observed at the *p21* promoter (Fig 3D), we tested promoter histone modifications for all the differentially expressed genes in HT1080 cells with long/short telomeres. For all the 11 genes where telomere elongation resulted in activation (Fig 4A) enrichment in activation histone modifications (either H3K4me1 or H3K4me3, or both) and/or depletion in the levels of the repressor modification H3K27me3 was found in HT1080-LT relative to HT1080 cells (Fig 5A–5C). On the other hand, in case of two genes down-regulated on telomere elongation—*PDGFRβ* and *WRNIP1—*activation histone marks H3K4me1 and H3K4me3 were either significantly depleted (*WRNIP1*) or the repressor mark H3K27me3 was enriched (*PDGFRβ*) within promoters in HT1080-LT compared to HT1080 cells. Taken together, in all the 13 genes differentially regulated on telomere elongation (Fig 4A), which also had reduced promoter TRF2 occupancy in HT1080-LT cells (Fig 1H), we found altered activation and/or repressor histone modifications within promoters to be consistent with TRF2-mediated activation or repression of the gene.

TRF2 silencing in HT1080 cells resulted in modifications in H3K4me1, H3K4me3 and H3K27me3 profiles on gene promoters (S8A–S8C Fig) that was largely consistent with promoter modifications observed in HT1080-LT relative to HT1080 cells (Fig 5). The histone modifications were also consistent with the differential gene expression on TRF2 silencing in HT1080 cells (Fig 1C). We further checked occupancy of REST at the 13 promoters that were sensitive to telomere length and TRF2 occupancy (in addition to *p21*). Eight out of the 13 promoters had REST occupancy (S9A Fig) and in case of 6 (of the 8) promoters REST occupancy was altered in HT1080-LT cells with elongated telomeres (S9B Fig).

## Discussion

Here we show that transcription of genes remote from telomeres depends on whether telomeres are short or elongated. This is mediated through non-telomeric TRF2 binding. Our results demonstrate that occupancy of TRF2 at telomere-distal gene promoters was relatively depleted when telomeres were long–consequently, TRF2-mediated transcription was affected. Notably, the epigenetic state of *p21* and many other promoters was dependent on telomere length, in a fashion consistent with TRF2-mediated up or down regulation of the gene.

### Telomere-sequestration and partitioning of TRF2 between telomeric and non-telomeric sites

**Fig 6:**
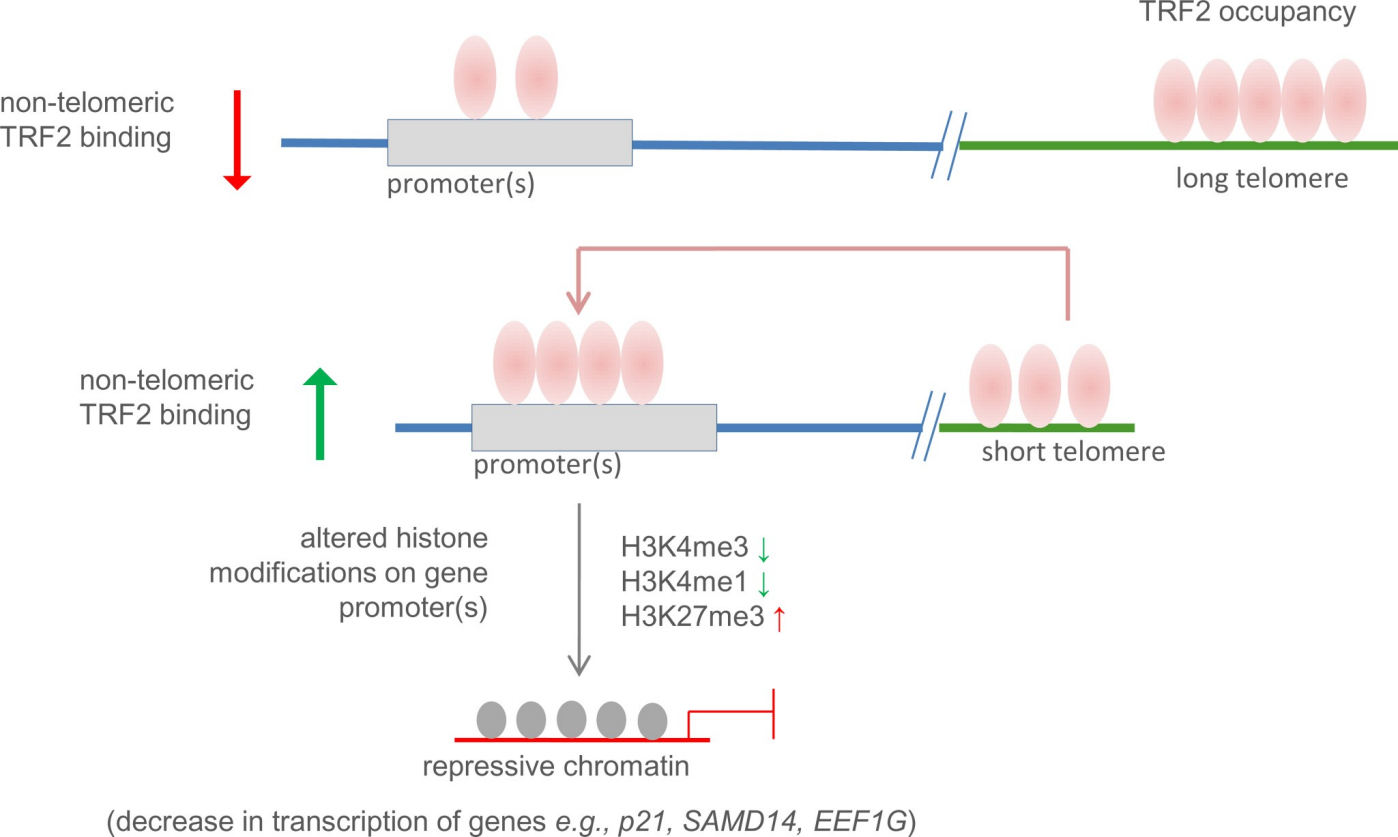
Telomere sequestration-partition model–non-telomeric versus telomeric TRF2 binding in cells with long vis-à-vis short telomeres. In cells with long telomeres increase in telomere-bound TRF2 restricts non-telomeric TRF2 occupancy. On the other hand, in cells with short telomeres, more TRF2 is available for binding at non-telomeric promoter sites. Increased TRF2 binding at promoters in cells with short vis-à-vis elongated telomeres result in altered chromatin histone modifications and influence transcription.

TRF2 binding at non-telomeric sites, particularly the extent of occupancy being dependent on state of telomeres (long/short) appears to be a key factor. As noted earlier, telomere-bound TRF2 was enhanced in cells with long telomeres[32]. However, total chromatin-bound TRF2 remained largely unaltered in cells with short versus long telomeres (in both HT1080 and MRC5 cells–S2E Fig and S3F Fig). Therefore, we postulated, enhanced telomeric TRF2 binding in cells with long telomeres may deplete TRF2 from non-telomeric sites. In contrast, in cells with short telomeres non-telomeric sites are likely to have more TRF2 occupancy relative to long telomeres. This was the case in both HT1080 and MRC5 cells (Fig 1H and Fig 2E). These argue for a model where TRF2 occupancy is partitioned between telomeric and non-telomeric sites. As a result, telomeric sequestration of TRF2 in cells with long telomeres restricts TRF2 binding at non-telomeric sites (Fig 6).

The number of TRF2 molecules/cell was reported to be ~50000 to 140000 depending on the cell type [38]. For HT1080 cells (mean telomere length of ~4.5 Kb[34,35]) ~69000 telomeric TRF2 binding sites are expected (S1 Text), which appears to be in the range of estimated TRF2 molecules/cell. Upon telomere elongation (to ~10 Kb) about 2-fold more TRF2 molecules are likely to be required for telomeric binding. However, TRF2 levels remained roughly similar in HT1080/HT1080-LT cells (S2D Fig). The affinity of TRF2 for telomeric/non-telomeric sites was also similar (S10 Fig). Despite this, we observed telomere-bound TRF2 increases in cells with longer telomeres consistent with an earlier report[32]. A possible explanation could be that TRF2 binds to telomeres as part of a larger complex with TRF1, RAP1, TIN2 and POT1[9,18,38–41]. In addition, RAP1 and TIN2 enhance association of TRF2 to telomeres[41,42]. This, along with the increased number of telomeric TRF2 binding sites in cells with elongated telomeres might help sequester more TRF2 to the telomeres.

Furthermore, TRF2 was primarily chromatin-bound in HT1080 and MRC5 cells (S2E Fig, S3F Fig), as also noted earlier[23]. Nucleoplasmic TRF2, though several folds lower than chromatin-bound, remained largely invariant in HT1080/HT1080LT cells (S2E Fig). In MRC5 cells, however, TRF2 in the nucleoplasm increased by ~15%, a small but reproducible observation (S3F Fig). It is not known if this is biologically significant but interaction of TRF2 with nucleoplasmic proteins such as lamin[29] and other nucleolar factors[43] has been reported. Further, nuclear lamin A was noted to be positively correlated with telomere length[44,45]. Therefore, it is possible that the higher nucleoplasmic TRF2 in telomere elongated MRC5 OF cells might be due to more lamin-bound TRF2.

### Promoter epigenetic changes are related to telomere length

Decrease in non-telomeric promoter TRF2 occupancy when telomeres are long induces, in most cases studied here, permissive chromatin (enriched H3K4me1/me3 and reduced H3K27me3 modifications; Fig 5A–5C, Fig 6). TRF2-mediated recruitment of the histone modification factors REST/LSD1 resulting in repression of *p21* was noted earlier[28]. Based on this, loss of TRF2 binding in cells with elongated telomeres gave reduced REST/LSD1 occupancy at the *p21* promoter (Fig 3C). As a result, active histone modifications at the *p21* promoter increased (and repressor modifications decreased) in cells with long vis-à-vis short telomeres (Fig 3D). In addition to *p21*, 8 of the 13 other promoters (sensitive to TRF2 occupancy and telomere length) had REST occupancy. In 6 (of the 8) promoters binding of REST decreased in HT1080-LT cells with elongated telomeres (S9A and S9B Fig) consistent with the histone modifications (Fig 5A–5C). It is possible that at the other promoters studied here histone changes result through chromatin modifications factors engaged in TRF2-dependent or independent ways. Although further work will be required to fully decipher the underlying mechanisms behind telomere-dependent distal promoter modifications and gene expression, these results provide early mechanistic support for our observations.

### Telomere length and genome-wide gene expression

Telomere-length dependent expression of several genes spread across the genome, and in two distinct cell types, observed here suggested transcriptome-wide changes may be linked to telomeres. To test we analyzed two independent microarray datasets[33,46] from short or elongated telomeres derived from isogenic cell lines. Isogenic background was necessary to limit confounding results from cell type-specific variations. In human pancreatic cancer PC-3 cells[46] (telomere elongation within tumors developed in mouse xenografts) analysis of microarray data across four replicates showed 1149 (out of 1461, ~78%) significantly differentially expressed genes (2-fold, p<0.05) were located beyond 10 Mb of telomeres (S11 Fig). Similarly, analysis of telomere-elongated versus control myoblast cells^33^ revealed >80% of the differentially regulated genes were distal to telomeres (2-fold, p<0.05; S11 Fig). These support our results obtained from a selected number of genes suggesting telomere length-dependent expression of genes are likely to be genome wide.

Looping of telomeres to interstitial sites, referred to as interstitial t-loops, mediated through TRF2 and lamin associations has been reported[29,47]. More recently, looping of the chromosome 5p telomere to the telomerase (*hTERT*) promoter 1.1 Mb from the telomeric end[48] was shown to result in presence of telomere-bound TRF2 at the *hTERT* promoter. Accordingly, TRF2 occupancy at the *hTERT* promoter was enriched in cells with long telomeres and reduced when telomeres were short and therefore less likely to have looping interactions. Both looping models (t-loop and *hTERT*-telomere) suggest enhanced non-telomeric TRF2 binding in case of long telomeres. In contrast, in the present study, we found loss in promoter TRF2 occupancy in cells with long telomeres. Conversely, for short telomeres, TRF2 occupancy at promoters was enriched. Since almost all the promoters were distal (tens of Mb mostly) to telomeres, this suggests TRF2 binding at non-telomeric sites may be distinct from the telomere looping mechanisms proposed earlier.

Telomere-dependent transcription of genes distal to telomeres shown here also appear distinct from the epigenetic phenomenon called telomere positioning effect (TPE) through which promoters close to the sub-telomeric regions remain silenced[49–51]. Extensively studied in the budding yeast TPE was reported to be primarily due to deacetylation of sub-telomeric nucleosomes by Rap1-mediated recruitment of SIR proteins[51]. *DUX4*, *C1S*, *ISG15* and *SORBS2* expression, located up to 10 Mb from telomeres, were also shown to depend on TPE but by a slightly different mechanism possibly involving telomeric looping to chromatin near these genes called TPE-OLD (over long distances)[33,52–54]. Accordingly, we noted TRF2 binding was enriched at the *ISG15* and *C1S* promoters in cells with relatively long telomeres (HT1080-LT and MRC5-OF cells; S12A and S12B Fig). This, again, was in contrast to loss of TRF2 occupancy found at the distal promoters in cells with elongated telomeres–and, therefore, unlikely to be due to telomere looping.

### Role of other telomeric factors in non-telomeric TRF2 function

Binding of TRF1 was reported at non-telomeric sites and in several instances in association with TRF2[25]. We checked for TRF1 binding within the 14 promoters where both change in TRF2 promoter occupancy and altered gene expression was observed in HT1080-LT cells with elongated telomeres. While TRF1 occupancy at the reported non-telomeric sites (HS3ST4 and CLIC6)[25] was retained, no significant TRF1 binding was observed within the 14 promoters in both HT1080 and HT1080-LT cells (S13 Fig).

Another shelterin factor RAP1 was also found to bind at non-telomeric sites[23,26]. Moreover, TRF2-RAP1 association has been reported[24,38,39,42,55]. Therefore, we asked whether the non-telomeric function of TRF2 reported here was RAP1 dependent. Expression of 14 genes that were sensitive to TRF2 was tested following RAP1 silencing. In all the 14 genes RAP1 silencing did not significantly affect TRF2-mediated expression (S14 Fig).

The consensus TRF2 binding site identified by the motif search algorithm MEME[56] in the promoters studied here showed a G-rich motif (S15 Fig). Though constructed from relatively few promoters, presence of the GGG trimer residues was consistent with the consensus TTAGGG motif within interstitial TRF2 binding sites reporter earlier[25]. Association with interstitial TTAGGG motifs was also reported for the shelterin protein RAP1 based on ChIP-seq[23]. For both–TRF2 and RAP1 –extra-telomeric binding, including RAP1-mediated genome wide transcription changes, was reported[23]^,^[25]. However, whether extra-telomeric binding (and expression changes) was influenced by telomere length was not tested.

With relative increase in p21 levels in cells with elongated telomeres, we did not notice much difference in percentage of cells in different phases of cell cycle (S16 Fig). One explanation for this is that in cells with elongated telomeres enhanced levels of hTERT, which is known to induce cell proliferation[57], might counter the effect of *p21* expression. Moreover, although ageing primary cells with shortened telomeres have increased *p21* expression (and decreased proliferation/senescence) in case of cancer cells, *p21* was observed to promote proliferation and oncogenicity in several studies[58–62]. Therefore, further work will be required to understand how telomere elongation/shortening impacts TRF2-mediated *p21* expression and resultant proliferation/senescence.

Following telomere replication during S phase, TRF2 is recruited to the newly formed telomeres to prevent telomeric DNA damage–consistent with the presence of TRF2 in S phase[63–65]. Therefore, it is possible that during S phase redistribution of TRF2 binding takes place as telomere length changes in the cells that were used in the present studies. However, further experiments will be required to test this.

In summary, our results show evidence of the telomeric shelterin protein TRF2 regulating expression of genes distal to telomeres in a telomere length-dependent way. While gene regulation by telomeric factors was reported, whether long or short telomeres had any impact on gene transcription at distances remote from telomeres was not studied[23,28,33]. Although based on a selected number of genes, our findings describe involvement of telomeres, in a mechanistic way through telomere-binding proteins such as TRF2. In addition, we observed epigenetics and transcriptional changes across the genome that had not been reported previously. A more complete understanding of this new regulatory mechanism of telomere binding proteins, might lead to an improved understanding of the molecular processes of how telomeres impact cellular physiology, particularly in ageing and cancer.

## Material and methods

### Cell lines, media and culture conditions

HT1080 fibrosarcoma cell line was purchased from the NCCS, Pune. Immortalized MRC5 cells were received as a gift from NII, New Delhi. HT1080, MRC5 cells and corresponding telomere elongated cells were maintained in Modified Eagle’s medium (MEM) supplemented with 10% Fetal Bovine Serum (FBS). All cultures were grown in incubators maintained at 37°C with 5% CO_2_.

### Flow-FISH

The Flow-FISH assay for telomere length detection was performed using DAKO Telomere PNA Kit/FITC codeK5327. Manufacturer’s’ guidelines were followed for assays.

### ChIP (Chromatin Immunoprecipitation)

ChIP assays were performed as per protocol provided by Upstate Biotechnology with modifications as suggested in Fast ChIP protocol. ChIP assays were performed using anti-TRF2 antibody (Novus Biologicals NB110-57130), anti-REST (Millipore), ani-LSD1 (CST), anti-H3K4me1, anti-H3K4me3, anti-H3K27me3 (Abcam). anti- TRF1 (TRF 78 Santa-Cruz),Anti-Rabbit IgG/Anti-mouse IgG was used for isotype control in all cell lines.

### TRF2 and RAP1 silencing

HT1080 cells/ MRC5 cells were transfected with TRF2 siRNA oligonucleotides (synthesized from Eurogenetics Pvt. Limited) /RAP1 pooled siRNA (Santacruz) using lipofectamine 2000 (Invitrogen) transfection reagent according to manufacturer’s instructions. Silencing was checked after 48 hr of transfection. Pooled SCR siRNA was used as control.

### Transfections

TRF2 WT (myc/DDK-tag), hTERT (Flag-tagged) and hTR cDNA cloned in mammalian expression vector pCMV6 was transfected into HT1080 cells that were 60% confluent using Lipofectamine 2000 transfection reagent (following the manufacturers’ protocol 2 μg of plasmid was used for transfection in a 35 mm well for each case. Expression was checked after 48 hr of transfection.

### Luciferase assay

Plasmid (pGL4.73) containing a CMV promoter driving Renilla luciferase was co-transfected as transfection control for normalization. After 48h, cells were harvested and luciferase activities of cell lysate were recorded by using a dual-luciferase reporter assay system (Promega).

### Real time PCR

Total RNA was isolated using TRIzol Reagent (Invitrogen, Life Technologies) according to manufacturer’s instructions. Relative transcript expression level for genes was measured by quantitative real-time PCR using SYBR Green form Takara.

### Dot blot analysis

For dot blot analysis, Genomic/ ChIP DNA was denatured at 95°C and dot blotted on N+ hybond membrane (Amersham) in pre-wetted in 2X SSC buffer. Rapid-Hyb buffer (Amersham) was used for blocking and hybridization as per manufacturer’s protocol.

### Chromatin and nucleoplasm fractionation assay

Chromatin fractionation assay was carried out as described earlier[66].The nuclear proteins are extracted by allowing cells to swell in hypotonic buffer and then disrupting the cells this is followed by removal of cytoplasmic fraction and using various combinations of low and high salt buffers nuclear proteins are released from nuclei.

### Western blotting

For western blot analysis, protein lysates were prepared by suspending cell pellets in 1X cell culture lysis buffer (Promega). Protein was separated using 12% SDS-PAGE and transferred to polyvinylidene difluoride membranes (Immobilon FL, Millipore). Primary antibodies- anti-TRF2 antibody (Novus Biological), anti-p21antibody (Cell signaling technology) and anti-β-actin/anti-GAPDH antibody (Sigma), anti-H2A (abcam). Secondary antibodies, anti-mouse and anti-rabbit alkaline phosphatase conjugates were from Sigma. The blot was finally developed by using Thermo Scientific Pierce NBT/BCIP developing reagents.

### TRAP assay for Telomerase activity

The assays were performed using TELO TAGG kit from ROCHE with adherence to manufacturer’s protocol. TRAP assay was performed using TeloTTAGG PCR ELISA kit from ROCHE catalog no.-11854666910. In this assay test cell lysate was used as the source for telomerase is provided PCR conditions allowing telomerase activity on biotinylated TS template. This reaction is followed by overall amplification. The amplified product is quantified by ELISA using Anti-DIG POD antibody performed on Streptavidin coated plate provided within the kit.

### Protein purification

Recombinant TRF2 was purified following expression in *E*. *coli*. Briefly, transformed cells were inoculated into 5 ml culture with Ampicillin (100 μg/ml) at 37°C overnight in a shaker incubator. 1 ml culture was inoculated with 500 ml fresh LB/Ampicillin and allowed to grow till OD of 0.6–0.8 units (at 600 nm wavelength). Following induction with 0.1 mM final concentration overnight at 18°C the culture was pelleted and sonicated in lysis buffer. 200 ul his-pure nickel NTA beads (Thermo Scientific) were added and incubated at 4°C on a rotatory shaker. Beads were washed consecutively with a 20 ml solution of 20–60 mM imidazole, and protein was eluted with 4 ml of 250 mM imidazole solution. Protein was concentrated along with buffer exchange to remove imidazole using Millipore 15 ml, 30 KDa concentrator columns. Purified protein was quantified by the BCA method (Thermo scientific BCA kit).

### ELISA assay

384-well streptavidin coated pre-blocked plates from Thermo Scientific (Pierce) were used for ELISA assay. Biotinylated oligonucleotides (IDT) were diluted to 5 picoM in 1X PBST buffer and loaded into each well, incubated at 37°C for 2 hours and washed 3 times with 1X PBST buffer. Purified TRF2 diluted in 1X PBST buffer was incubated with oligonucleotides for 2 hours at 4°C, washed 5 times with 1X PBST buffer, anti-TRF2 antibody (Novus NB110-57130) was added 1:1000 dilution (30 ul per well) and incubated for 1 hr at room temperature. Wells were washed five times with 1X PBST, 10 ul BCIP/NBT substrate was added to each well and absorbance was recorded at 610 nm using TECAN multimode reader. Two controls were used in ELISA assay to subtract background binding of antibody and protein. Protein negative control: except TRF2 protein all other reagents were added to determine the background binding of antibodies. Oligonucleotide negative control: except oligonucleotide, all other reagents along with increasing concentration of protein were added to determine background binding of the protein. The absorbance obtained from control wells were used for normalization and data analyzed using GraphPad Prism7.

### BrdU Incorporation assay

Assay was performed using FITC BrdU Flow Kit from BD Pharmingen using manufacturer provided protocol.

## Supporting information

S1 Fig A. TRF2 occupancy at sites with no reported TRF2 binding (negative control loci) in HT1080 and MRC5 cells.
B. Western blots to confirm TRF2 silencing in HT1080 and MRC5 cells
C. Expression of negative control genes upon TRF2 silencing
Error bars represent ± SD from three independent experiments.
(TIF)

S2 Fig **A**. Telomere elongation in HT1080-LT cells was confirmed by Flow-FISH using fluorescently labeled telomere-specific probes. Relative telomere length was quantified by three independent Flow-FISH experiments. Error bars indicate ± SD from three independent experiments; significance was tested by paired T-test -* <0.05; **<0.01.
**B. HT1080-LT cells harbor increased telomeric DNA**. Dot blot showing telomeric probe intensity, quantification in the frame on right (normalized to Alu) for HT1080-LT and HT1080 cells; error bars indicate ± SD from two independent experiments.
**C. HT1080-LT cells have higher *hTERT* / *hTERC* expression and telomerase activity**. HT1080-LT cells with increased *hTERT* and *hTERC* levels (determined by qRT-PCR) and telomerase activity as determined by quantitative real-time TRAP (telomerase repeat amplification). Error bars indicate ± SD from three independent experiments. Significance was tested by paired T-test -* <0.05; **<0.01
**D. HT1080-LT cells have higher telomeric TRF2 occupancy**. Dot blot showing telomeric probe intensity in 1% Input, ChIP and Mock(IgG) samples in HT1080 and HT1080-LT cells.
**E. TRF2 in nucleoplasm fraction and chromatin bound-TRF2 similar in HT1080 and HT1080-LT cells**. Nuclear TRF2 levels were comparable in chromatin as well as nucleoplasm fraction in HT1080-LT and HT1080 cells. quantification in the frame on right (normalized to beta actin) for HT1080-LT and HT1080 cells; error bars indicate ± SD from two independent experiments.
**F. TRF2 in whole cell lysate was similar in HT1080 and HT1080-LT cells**.
Quantification in the frame on right (normalized to beta actin) for HT1080-LT and HT1080 cells; error bars indicate ± SD from two independent experiments.
(TIF)

S3 Fig **A. Scheme showing steps followed in generating cells with artificially elongated telomere**. Cells were treated with G-rich terminal repeats (GTR) [(TTAGGG)4 100 mM] oligonucleotides in serum free media. After the treatment, GTR containing media was removed after 6 hrs, followed by media wash twice and cells were grown for 24 hrs before splitting. Cycles were repeated to obtain cells with desired number of oligonucleotide feeding (OF).
**B. MRC5 oligo-fed cells with increased telomeric DNA**. Dot blot showing telomeric probe signal (normalized to Alu; quantification in the right frame) in MRC5-untreated and oligo-fed cells (OF7,OF14). Error bars indicate ± SD from two independent experiments.
**C**. MRC5 oligo-fed cells (OF7, OF14) show enhanced *hTERT* and *hTERC* (determined by qRT-PCR). Error bars indicate ± SD from three independent experiments; significance was tested by paired T-test -* <0.05; **<0.01.
**D**. Relative telomerase activity of MRC5-untreated and OF7 or OF14 cells determined by quantitative real time TRAP assay in three technical replicates.
**E. MRC5 oligo-fed cells have higher telomeric TRF2 occupancy**. Dot blot showing telomeric probe intensity in 1% Input, ChIP and Mock(IgG) samples MRC5-untreated and oligo-fed cells (OF7,OF14).
**F-G**. TRF2 in nucleoplasm fraction, chromatin bound-TRF2 (F) and whole cell lysate(G) in MRC5-untreated and OF7 or OF14 cells. Quantification in the frame on right (normalized to beta actin); error bars indicate ± SD from two independent experiments.
(TIF)

S4 Fig Telomerase over expression does not change promoter TRF2 occupancy and p21 regulation. Upon transient overexpression of *hTERT* and *hTERC* in HT1080 cells, there was no significant change in TRF2 occupancy at the *p21* promoter and *p21* expression.
**A**. Over expression of *hTERT* and *hTERC* was confirmed by qRT PCR in HT1080 cells.
**B**. TRF2 occupancy on p21 promoter was checked in cells over expressing *hTERT* and *hTERC*
**C**. *p21* expression was checked in cells over expressing *hTERT* and *hTERC*
Error bars in all cases indicate ± SD from two independent experiments.
(TIF)

S5 Fig **A. REST ChIP** samples in HT1080 and HT1080-LT cells tested for occupancy at *Synapsin* promoter (positive control for REST ChIP); *p21* promoter and *CTCF* promoter (negative control locus for REST ChIP).
**B. LSD1 ChIP** samples in HT1080 and HT1080-LT cells tested for occupancy at *p21* promoter and *CTCF* promoter (negative control locus for LSD 1 ChIP).
Error bars indicate ± SD from three independent experiments; significance was tested by paired T-test -* <0.05; **<0.01.
(TIF)

S6 Fig A. Expression of TRF2 target genes in HT1080 cells with independent or combined *hTERT* and *hTERC* over expression
B. Over expression of *hTERT* and *hTERC* was confirmed by qRT PCR in HT1080 cells.
Error bars in all cases indicate ± SD from three independent experiments.; significance was tested by paired T-test -* <0.05; **<0.01.
(TIF)

S7 Fig **A**. Effect of TRF2 over expression on genes with telomere length-dependent TRF2 occupancy in HT1080-LT cells.
**B**. Comparison of gene expression in HT1080 and HT1080-LT cells following TRF2 over expression
**C**. Confirmation of TRF2 over-expression in HT1080 and HT1080-LT cells by qPCR.
Error bars indicate ± SD from three independent experiments; significance was tested by paired T-test -* <0.05; **<0.01.
(TIF)

S8 Fig Comparison of histone marks H3K4me1 (A), H3K4me3(B) and H3K27me3(C) in TRF2 silenced condition versus scrambled control in HT1080 cells. Error bars indicate ± SD from two independent experiments.
(TIF)

S9 Fig A. REST occupancy at gene promoters in HT1080 cells.
B. Comparison of REST occupancy at gene promoters in HT1080 and HT 080-LT cells.
Error bars indicate ± SD from two independent experiments.
(TIF)

S10 Fig Binding of recombinant TRF2 affinity with extra-telomeric binding sites within promoters of indicated genes and the telomeric sequence. (TIF)

S11 Fig Differential expression of genes in PC-3 tumors or myoblast cells with elongated telomeres relative to respective control cells reanalyzed from publicly available microarray data (Seimiya *et al*., 2013, Shay *et al*., 2014). Differential expression was analyzed using GEO2R package provided by Gene Entry Omnibus for both the datasets. For GSE41559, cells with long and short telomeres with basal TERT (four replicates each) was analyzed using two-fold change cutoff at p<0.05. For GSE99552, myoblast cells with long and short telomeres (three replicates each) was analyzed using two-fold change cutoff at p<0.05. Annotation of gene location was done using publicly available tool DAVID 6.8 (Huang *et al*, 2009) using Refseq genes mapped on to hg19 genome assembly.
(TIF)

S12 Fig TRF2 occupancy at sub-telomeric sites sensitive to telomere looping interactions. TRF2 occupancy at gene promoter sites reported for sub telomeric looping was checked HT1080 cells and HT1080-LT cells (A) and MRC5 OF cells (B).
Error bars indicate ± SD from three independent experiments. significance was tested by paired T-test -* <0.05; **<0.01.
(TIF)

S13 Fig TRF1 occupancy on gene promoters in HT1080 and HT1080-LT cells (top panel). Internal telomeric repeats within *HS3ST4* and *CLIC6* genes reported to bind TRF1 were used as positive control for TRF1 ChIP (bottom panel). Error bars indicate ± SD from three independent experiments.
(TIF)

S14 Fig **A**. Effect of RAP1 silencing on TRF2-mediated gene expression.
**B**. Confirmation of TRF2 over expression and RAP1 knockdown by qRT PCR
Error bars indicate ± SD from two independent experiments.
(TIF)

S15 Fig Motif search in ChIP PCR validated regions on target promoters. **A**. Consensus G-rich motif detected by MEME application for motif discovery within gene promoters validated for TRF2 binding
**B** Individual motif sequences, distance of motif from TSS and significance of motif detection as obtained from MEME for gene promoters with differential promoter TRF2 occupancy in cells with short vs long telomeres. (detected motif nearest to the TSS have been shown).
(TIF)

S16 Fig Distribution of cells in G1, S and G2/M phases (as percentage of total cells) in HT1080 and HT1080-LT cells as estimated by BrdU Incorporation assay. (TIF)

S1 Table TRF2 occupancy at interstitial gene promoters and their distance form telomere ends. TRF2 binding sites within 1500 bp upstream of transcription start sites (TSS) were validated by ChIP-qRT PCR.
(TIF)

S1 Text Expected number of telomeric TRF2 sites. Number of TRF2 molecules per cell was noted to be around 50000 to 140000 depending on the cell type (Takai et al. 2010).
TRF2 associates with two units of telomeric TTAGGG repeats as a dimer (Yang et al. 2011; Lim et al. 2017) suggesting a 6-mer binding motif per TRF2 molecule.
In case of HT1080 cells of mean telomere length of ~4.5 Kb (Chen et al. 2012; Pickett et al. 2009) this would result in ~69000 telomeric TRF2 binding sites.
4500 bp x46(chr) x2 (diploid) = 414000 bp
414000 bp / 6 (6-mer TRF2 binding site) = 69000 telomeric TRF2 binding sites.
(TIF)
