# Correction: Telomere length-dependent transcription and epigenetic modifications in promoters remote from telomere ends

**DOI:** 10.1371/journal.pgen.1009152

**Published:** 2020-10-22

**Authors:** Ananda Kishore Mukherjee, Shalu Sharma, Suman Sengupta, Dhurjhoti Saha, Pankaj Kumar, Tabish Hussain, Vivek Srivastava, Sumitabho Deb Roy, Jerry W. Shay, Shantanu Chowdhury

There is an error in affiliation 2 for authors Ananda Kishore Mukherjee, Shalu Sharma, Pankaj Kumar and Shantanu Chowdhury. The correct affiliation 2 is: Academy of Scientific and Innovative Research (AcSIR), Ghaziabad-201002, India.
