# Supplementary figures and images for: Telomere length-dependent transcription and epigenetic modifications in promoters remote from telomere ends
Source: PLoS Genet. 2018 Nov 15;14(11):e1007782. doi: 10.1371/journal.pgen.1007782 (PMC6264879; doi:10.1371/journal.pgen.1007782)

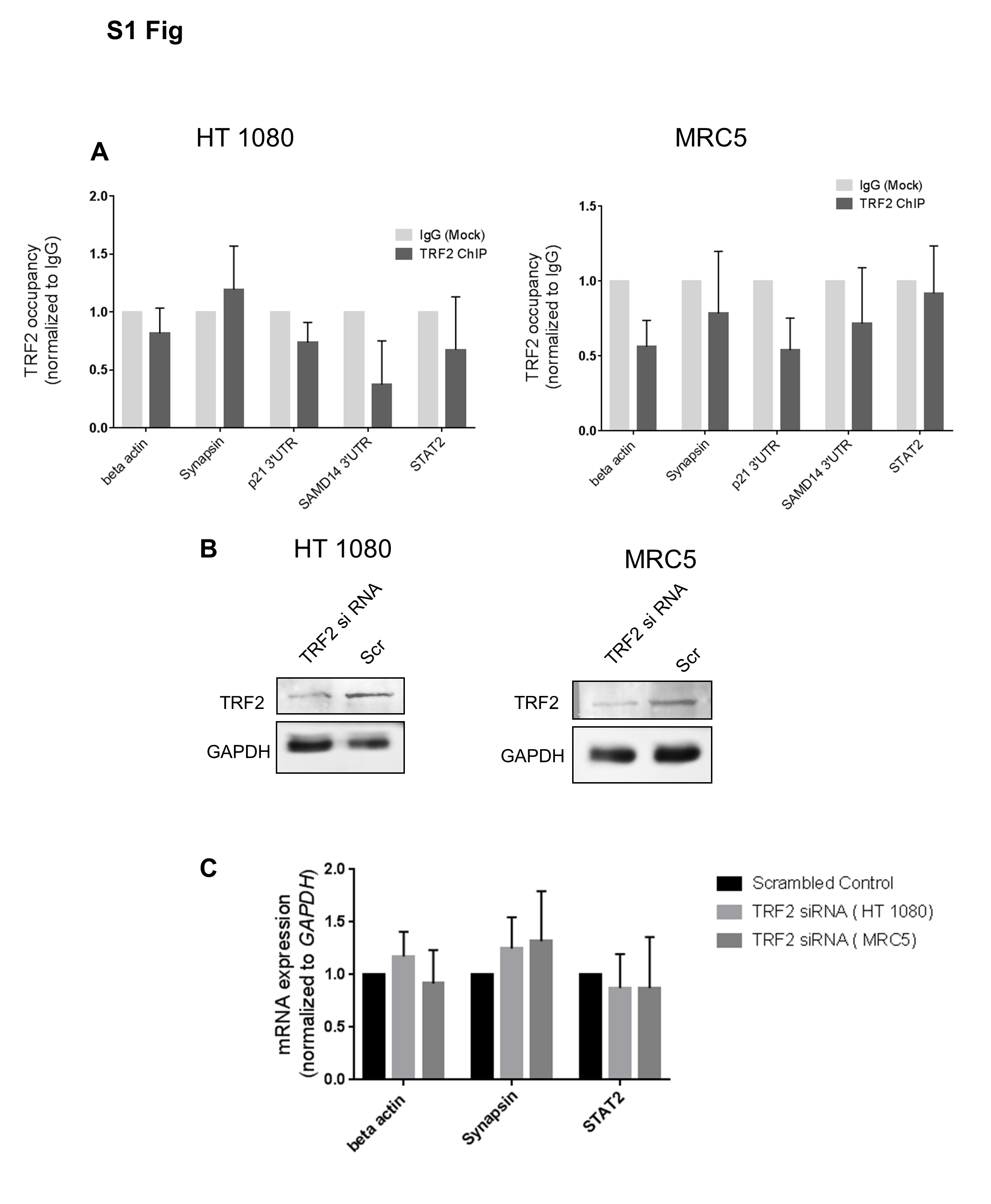

Supplement: S1 Fig — A. TRF2 occupancy at sites with no reported TRF2 binding (negative control loci) in HT1080 and MRC5 cells. B. Western blots to confirm TRF2 silencing in HT1080 and MRC5 cells C. Expression of negative control genes upon TRF2 silencing Error bars represent ± SD from three independent experiments. (TIF) [file pgen.1007782.s001.TIF]

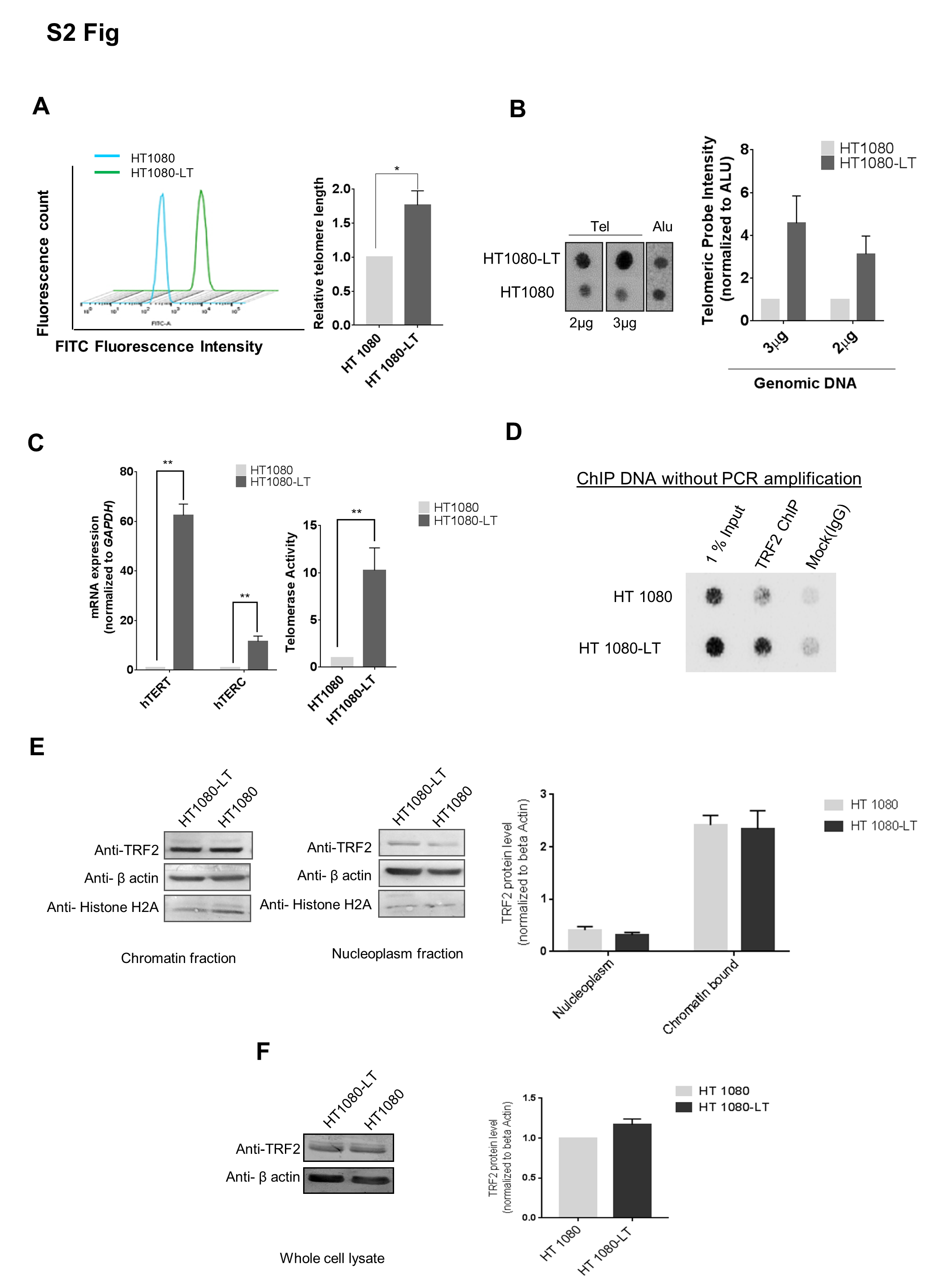

Supplement: S2 Fig — A. Telomere elongation in HT1080-LT cells was confirmed by Flow-FISH using fluorescently labeled telomere-specific probes. Relative telomere length was quantified by three independent Flow-FISH experiments. Error bars indicate ± SD from three independent experiments; significance was tested by paired T-test -* <0.05; **<0.01. B. HT1080-LT cells harbor increased telomeric DNA. Dot blot showing telomeric probe intensity, quantification in the frame on right (normalized to Alu) for HT1080-LT and HT1080 cells; error bars indicate ± SD from two independent experiments. C. HT1080-LT cells have higher hTERT / hTERC expression and telomerase activity. HT1080-LT cells with increased hTERT and hTERC levels (determined by qRT-PCR) and telomerase activity as determined by quantitative real-time TRAP (telomerase repeat amplification). Error bars indicate ± SD from three independent experiments. Significance was tested by paired T-test -* <0.05; **<0.01 D. HT1080-LT cells have higher telomeric TRF2 occupancy. Dot blot showing telomeric probe intensity in 1% Input, ChIP and Mock(IgG) samples in HT1080 and HT1080-LT cells. E. TRF2 in nucleoplasm fraction and chromatin bound-TRF2 similar in HT1080 and HT1080-LT cells. Nuclear TRF2 levels were comparable in chromatin as well as nucleoplasm fraction in HT1080-LT and HT1080 cells. quantification in the frame on right (normalized to beta actin) for HT1080-LT and HT1080 cells; error bars indicate ± SD from two independent experiments. F. TRF2 in whole cell lysate was similar in HT1080 and HT1080-LT cells. Quantification in the frame on right (normalized to beta actin) for HT1080-LT and HT1080 cells; error bars indicate ± SD from two independent experiments. (TIF) [file pgen.1007782.s002.TIF]

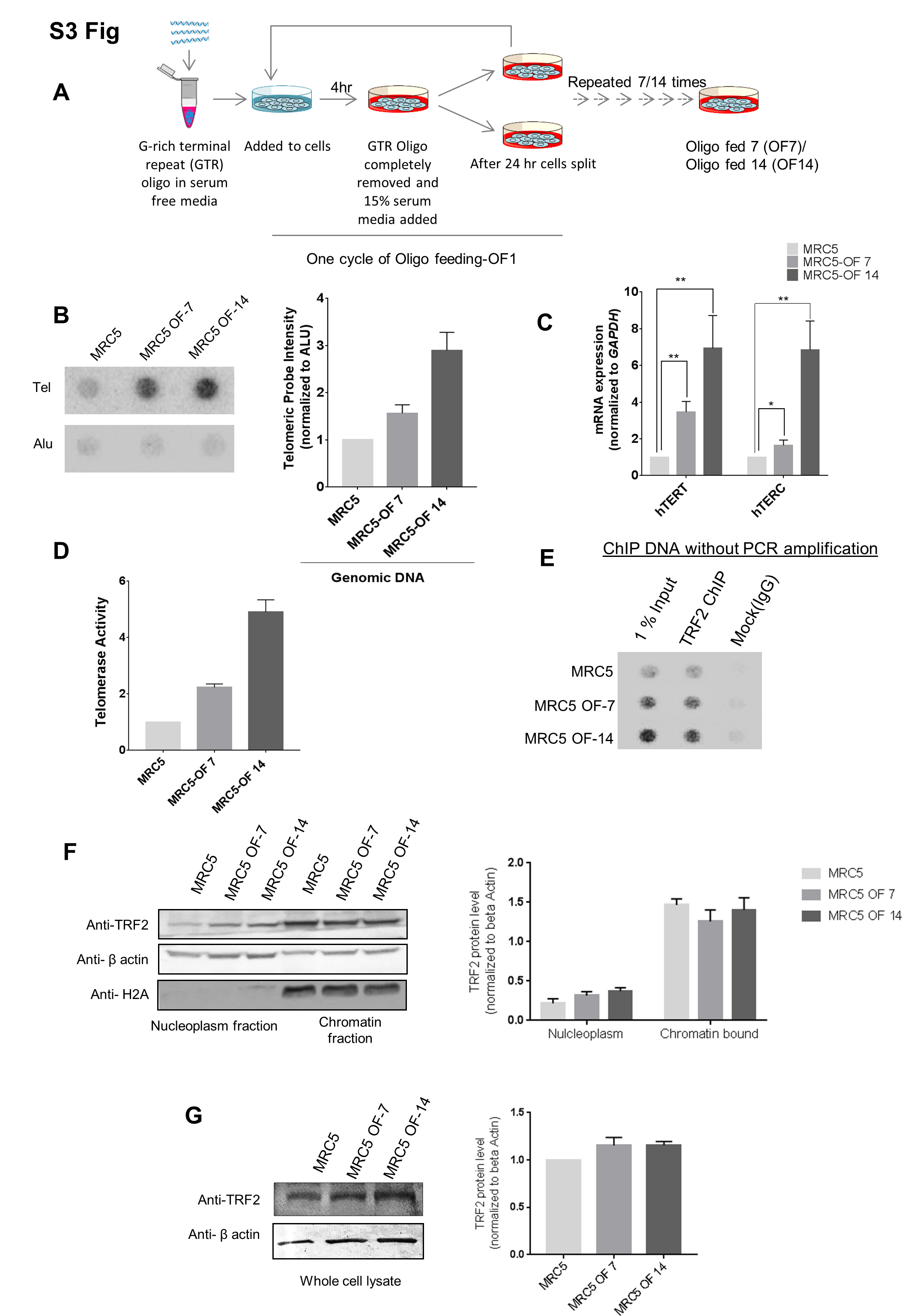

Supplement: S3 Fig — A. Scheme showing steps followed in generating cells with artificially elongated telomere. Cells were treated with G-rich terminal repeats (GTR) [(TTAGGG)4 100 mM] oligonucleotides in serum free media. After the treatment, GTR containing media was removed after 6 hrs, followed by media wash twice and cells were grown for 24 hrs before splitting. Cycles were repeated to obtain cells with desired number of oligonucleotide feeding (OF). B. MRC5 oligo-fed cells with increased telomeric DNA. Dot blot showing telomeric probe signal (normalized to Alu; quantification in the right frame) in MRC5-untreated and oligo-fed cells (OF7,OF14). Error bars indicate ± SD from two independent experiments. C. MRC5 oligo-fed cells (OF7, OF14) show enhanced hTERT and hTERC (determined by qRT-PCR). Error bars indicate ± SD from three independent experiments; significance was tested by paired T-test -* <0.05; **<0.01. D. Relative telomerase activity of MRC5-untreated and OF7 or OF14 cells determined by quantitative real time TRAP assay in three technical replicates. E. MRC5 oligo-fed cells have higher telomeric TRF2 occupancy. Dot blot showing telomeric probe intensity in 1% Input, ChIP and Mock(IgG) samples MRC5-untreated and oligo-fed cells (OF7,OF14). F-G. TRF2 in nucleoplasm fraction, chromatin bound-TRF2 (F) and whole cell lysate(G) in MRC5-untreated and OF7 or OF14 cells. Quantification in the frame on right (normalized to beta actin); error bars indicate ± SD from two independent experiments. (TIF) [file pgen.1007782.s003.TIF]

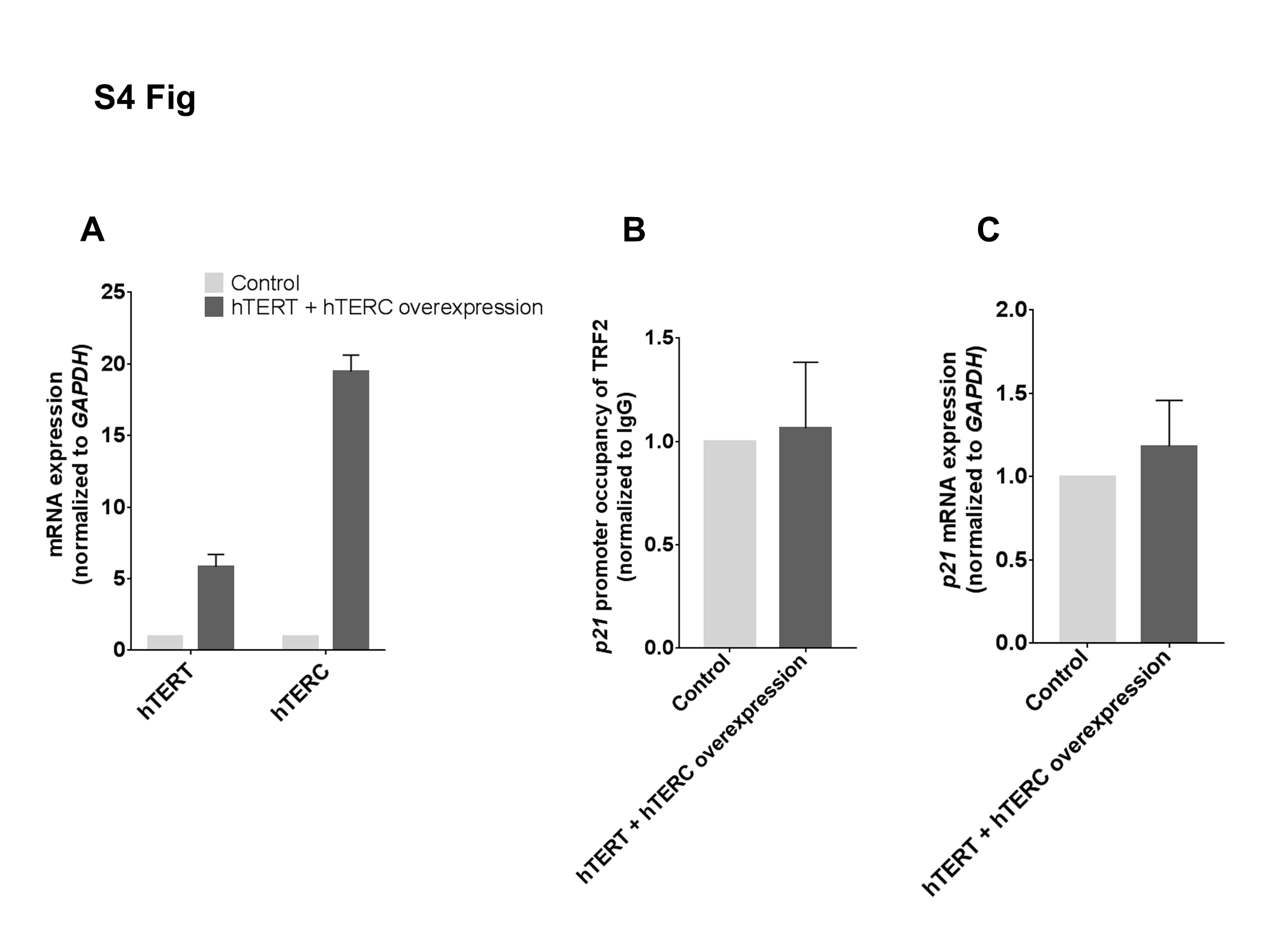

Supplement: S4 Fig — Upon transient overexpression of hTERT and hTERC in HT1080 cells, there was no significant change in TRF2 occupancy at the p21 promoter and p21 expression. A. Over expression of hTERT and hTERC was confirmed by qRT PCR in HT1080 cells. B. TRF2 occupancy on p21 promoter was checked in cells over expressing hTERT and hTERC C. p21 expression was checked in cells over expressing hTERT and hTERC Error bars in all cases indicate ± SD from two independent experiments. (TIF) [file pgen.1007782.s004.TIF]

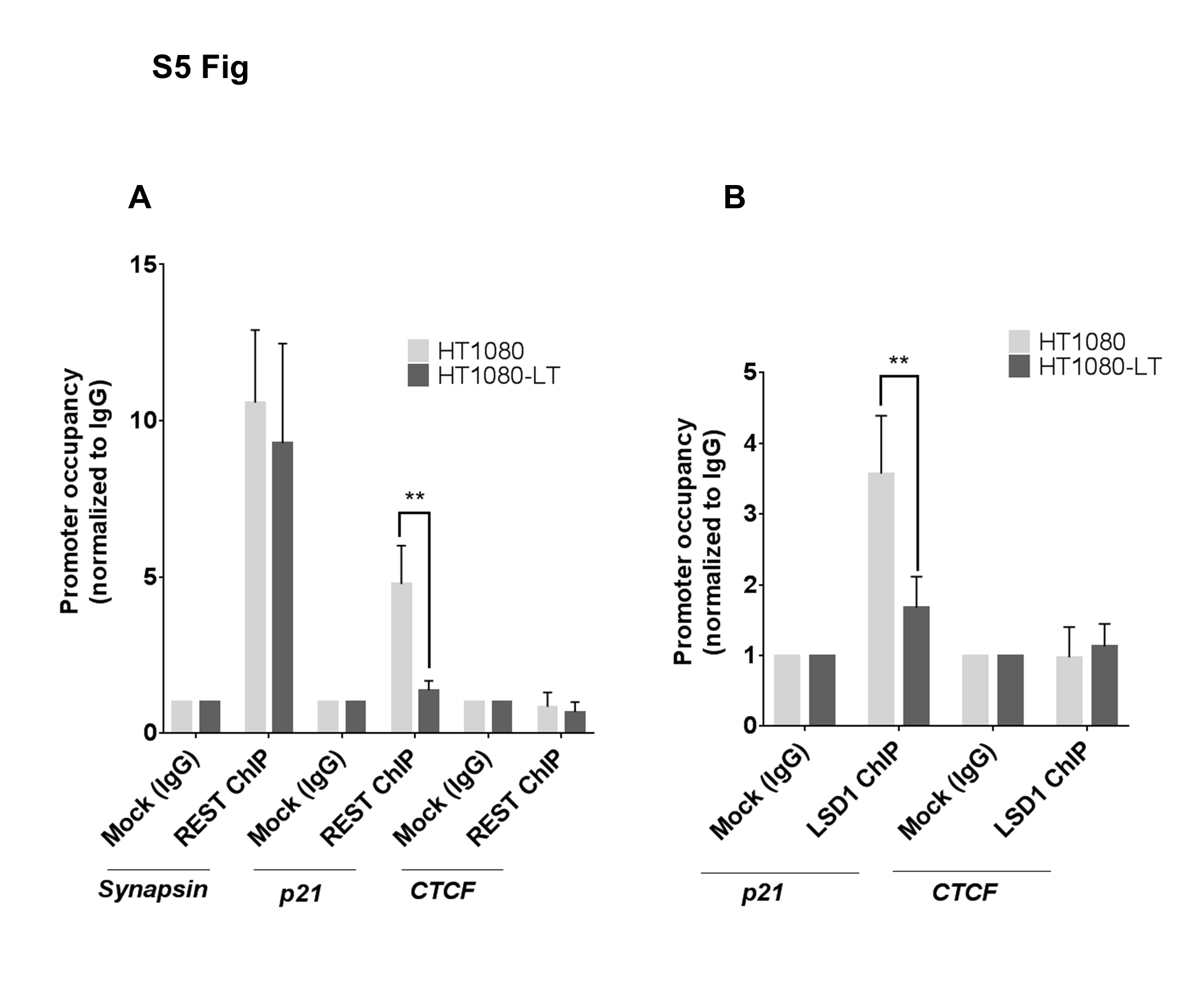

Supplement: S5 Fig — A. REST ChIP samples in HT1080 and HT1080-LT cells tested for occupancy at Synapsin promoter (positive control for REST ChIP); p21 promoter and CTCF promoter (negative control locus for REST ChIP). B. LSD1 ChIP samples in HT1080 and HT1080-LT cells tested for occupancy at p21 promoter and CTCF promoter (negative control locus for LSD 1 ChIP). Error bars indicate ± SD from three independent experiments; significance was tested by paired T-test -* <0.05; **<0.01. (TIF) [file pgen.1007782.s005.TIF]

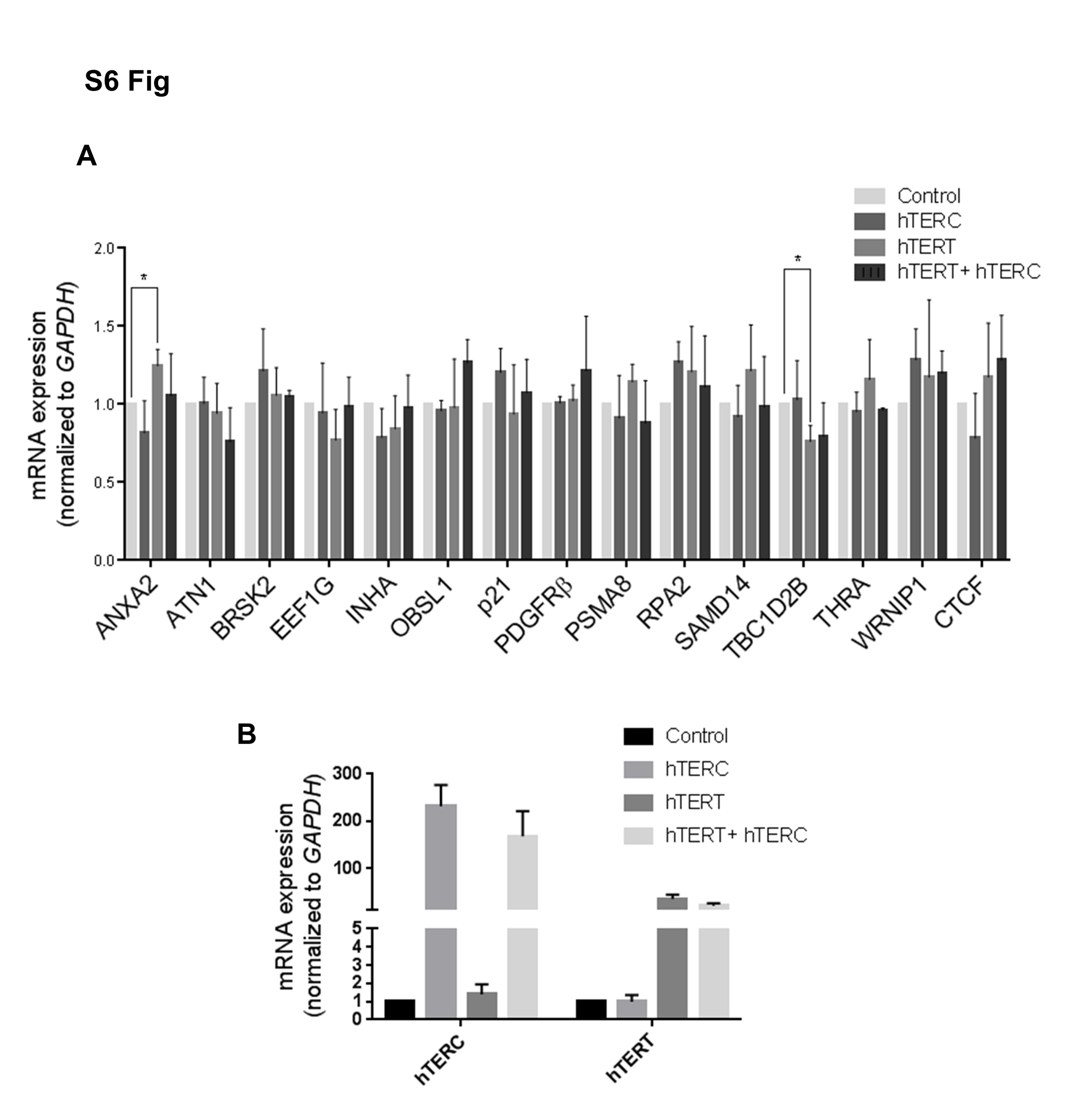

Supplement: S6 Fig — A. Expression of TRF2 target genes in HT1080 cells with independent or combined hTERT and hTERC over expression B. Over expression of hTERT and hTERC was confirmed by qRT PCR in HT1080 cells. Error bars in all cases indicate ± SD from three independent experiments.; significance was tested by paired T-test -* <0.05; **<0.01. (TIF) [file pgen.1007782.s006.TIF]

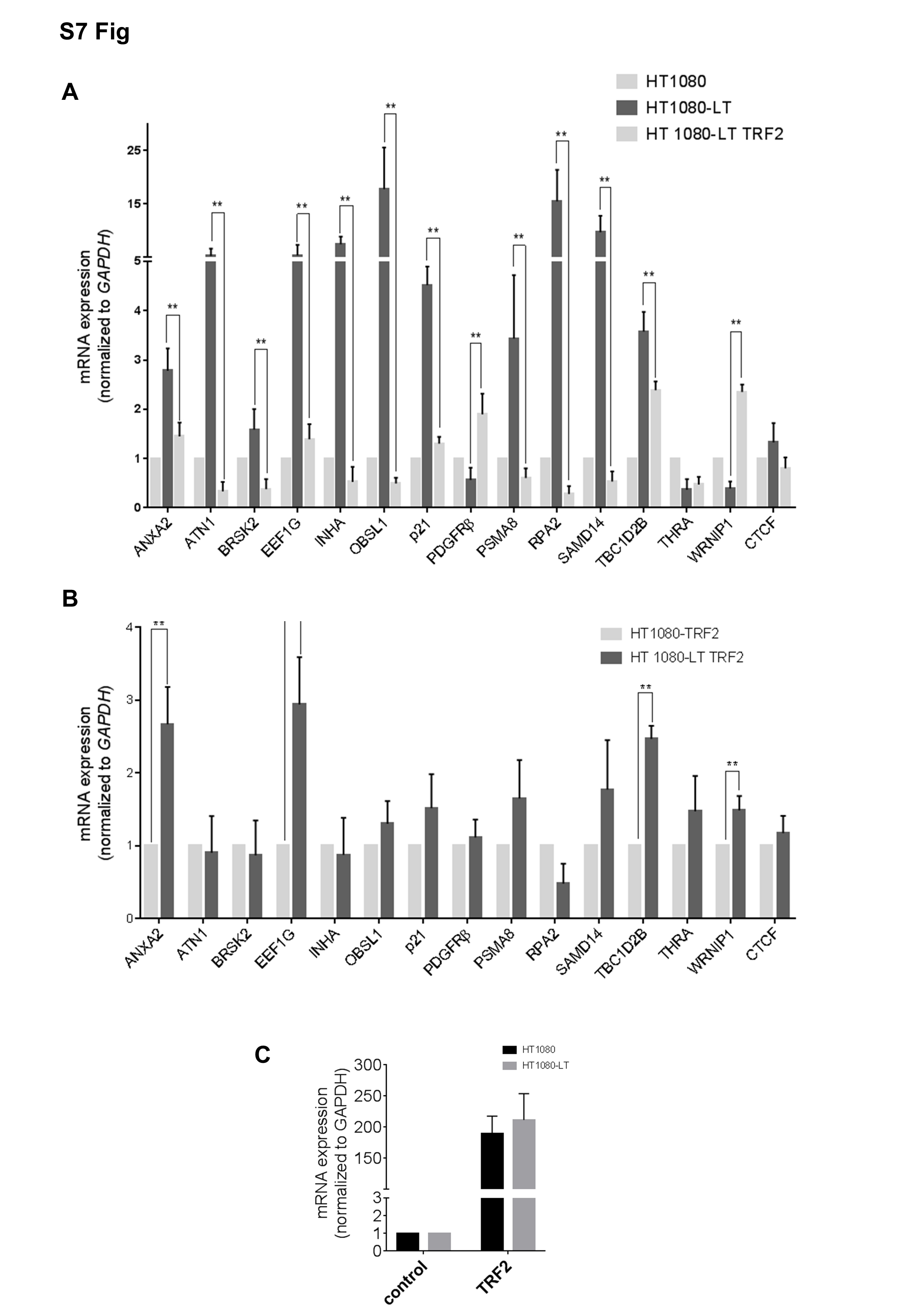

Supplement: S7 Fig — A. Effect of TRF2 over expression on genes with telomere length-dependent TRF2 occupancy in HT1080-LT cells. B. Comparison of gene expression in HT1080 and HT1080-LT cells following TRF2 over expression C. Confirmation of TRF2 over-expression in HT1080 and HT1080-LT cells by qPCR. Error bars indicate ± SD from three independent experiments; significance was tested by paired T-test -* <0.05; **<0.01. (TIF) [file pgen.1007782.s007.TIF]

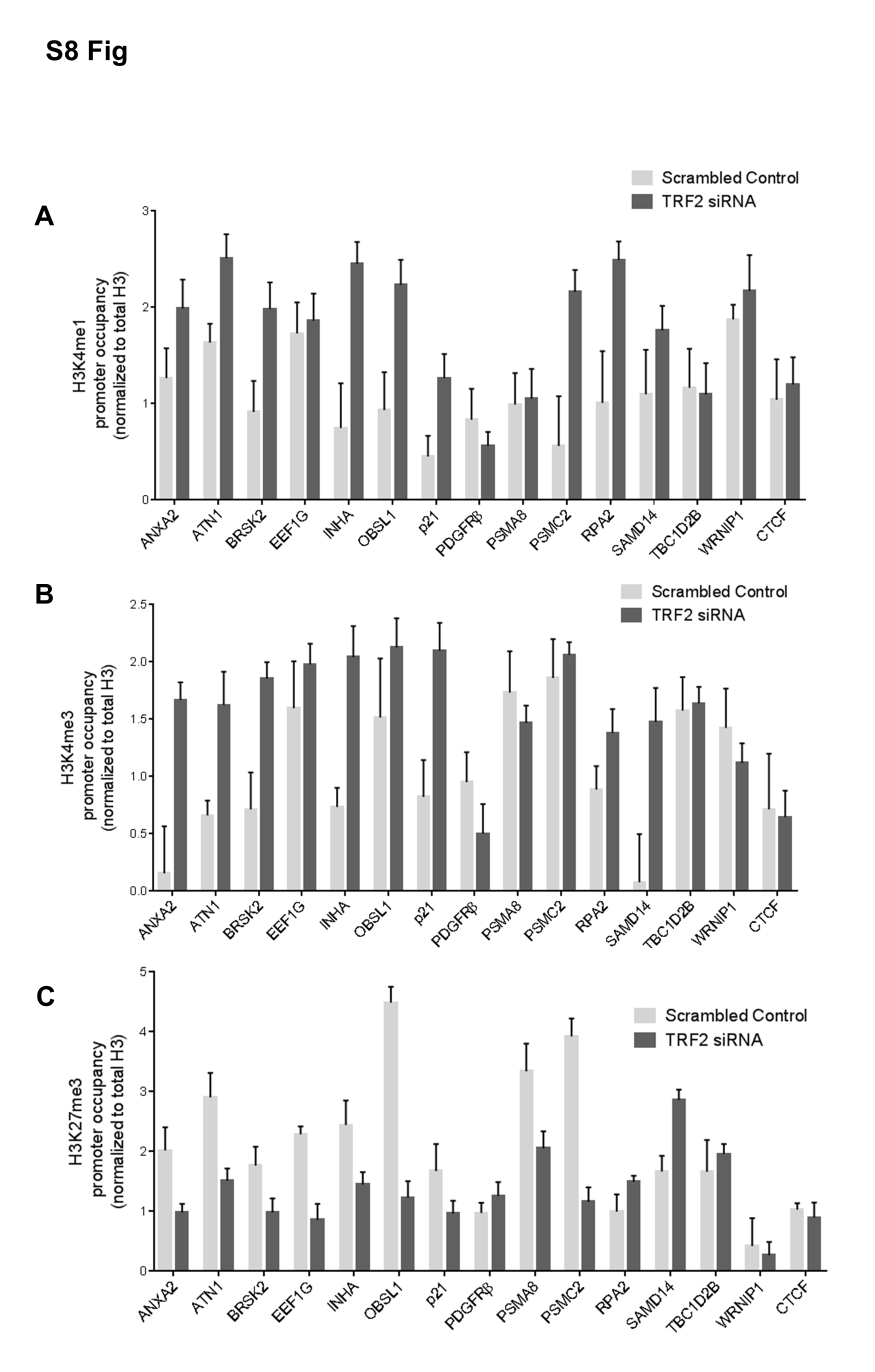

Supplement: S8 Fig — Comparison of histone marks H3K4me1 (A), H3K4me3(B) and H3K27me3(C) in TRF2 silenced condition versus scrambled control in HT1080 cells. Error bars indicate ± SD from two independent experiments. (TIF) [file pgen.1007782.s008.TIF]

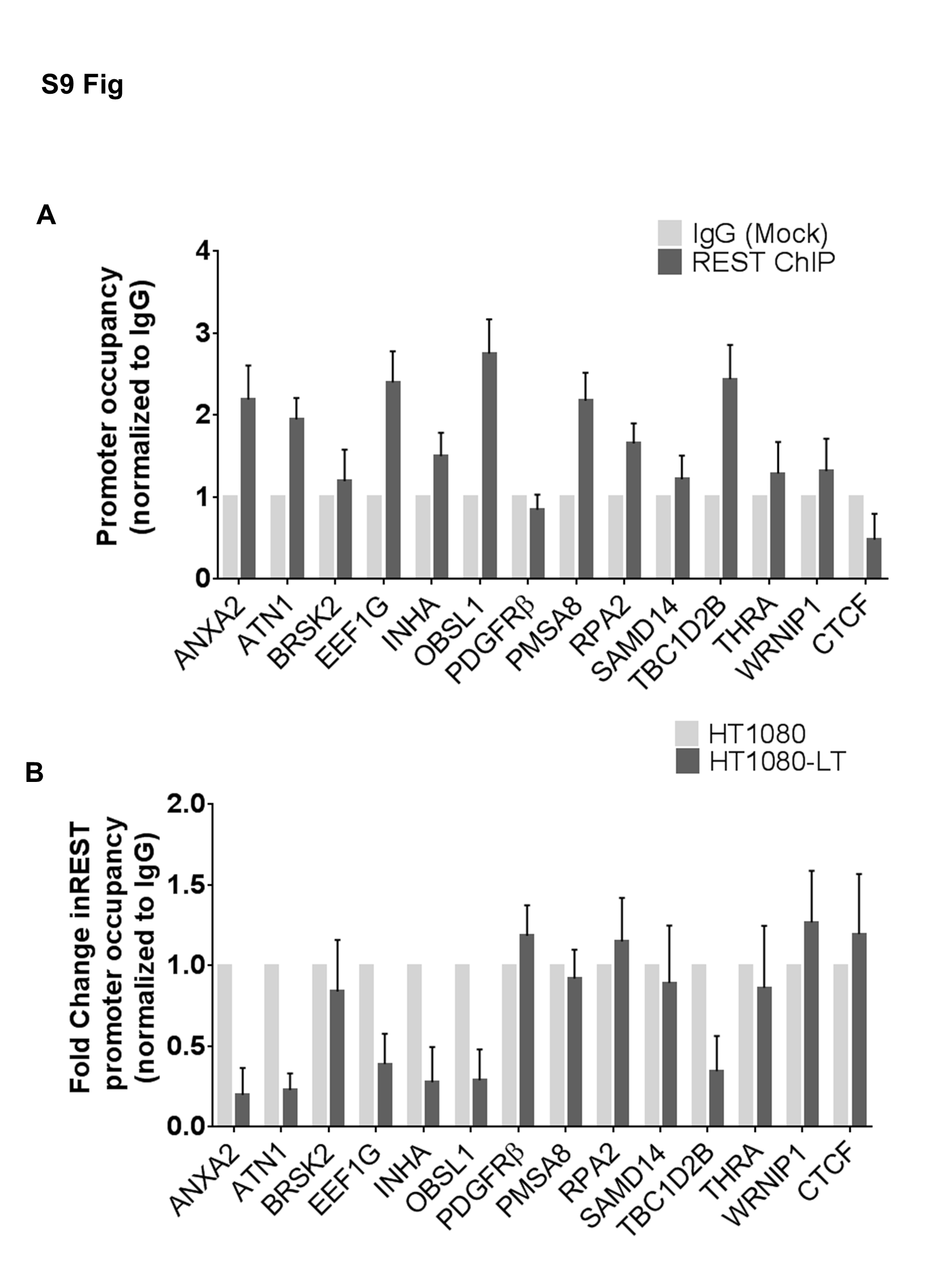

Supplement: S9 Fig — A. REST occupancy at gene promoters in HT1080 cells. B. Comparison of REST occupancy at gene promoters in HT1080 and HT 080-LT cells. Error bars indicate ± SD from two independent experiments. (TIF) [file pgen.1007782.s009.TIF]

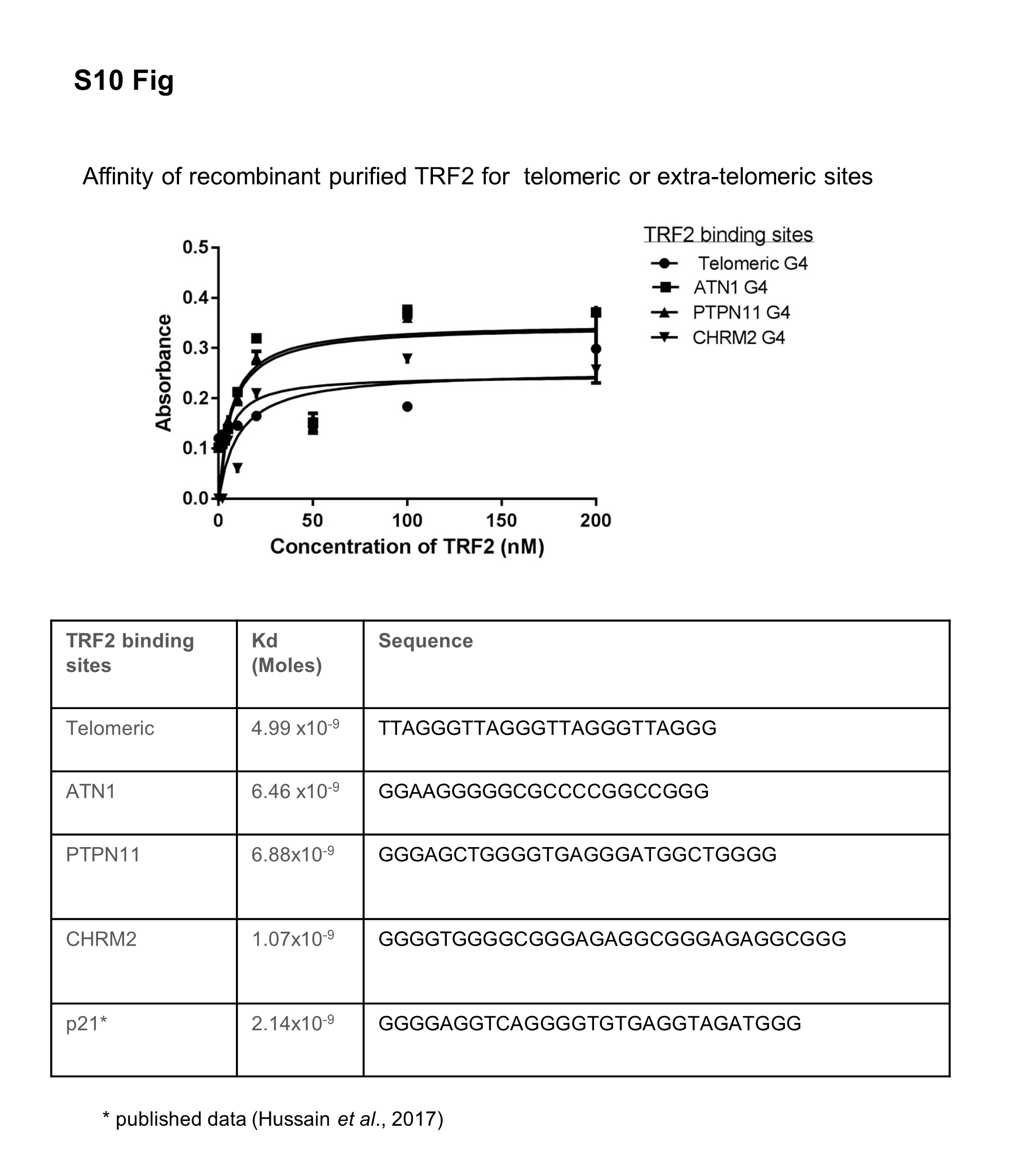

Supplement: S10 Fig — (TIF) [file pgen.1007782.s010.TIF]

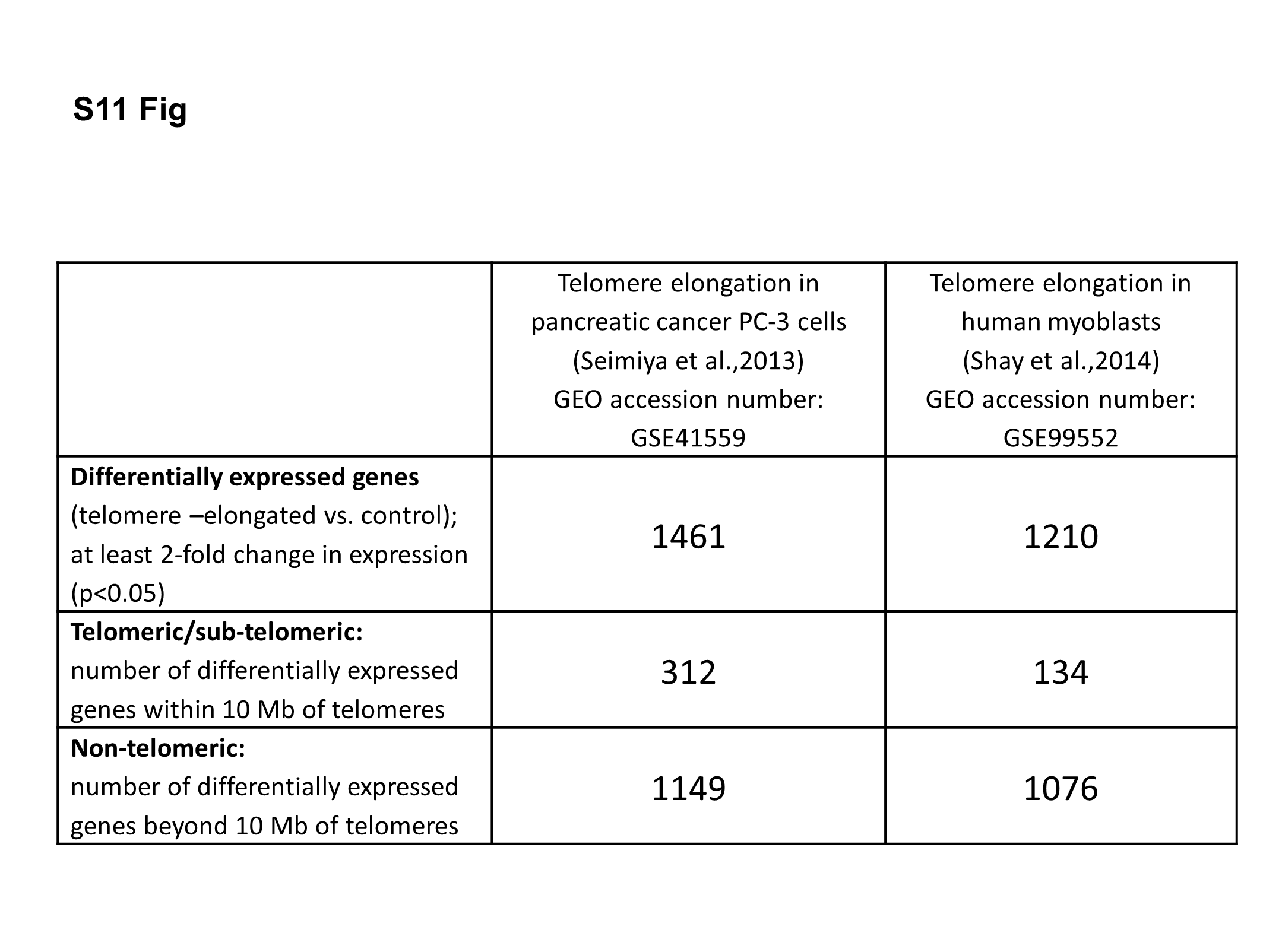

Supplement: S11 Fig — Differential expression was analyzed using GEO2R package provided by Gene Entry Omnibus for both the datasets. For GSE41559, cells with long and short telomeres with basal TERT (four replicates each) was analyzed using two-fold change cutoff at p<0.05. For GSE99552, myoblast cells with long and short telomeres (three replicates each) was analyzed using two-fold change cutoff at p<0.05. Annotation of gene location was done using publicly available tool DAVID 6.8 (Huang et al, 2009) using Refseq genes mapped on to hg19 genome assembly. (TIF) [file pgen.1007782.s011.TIF]

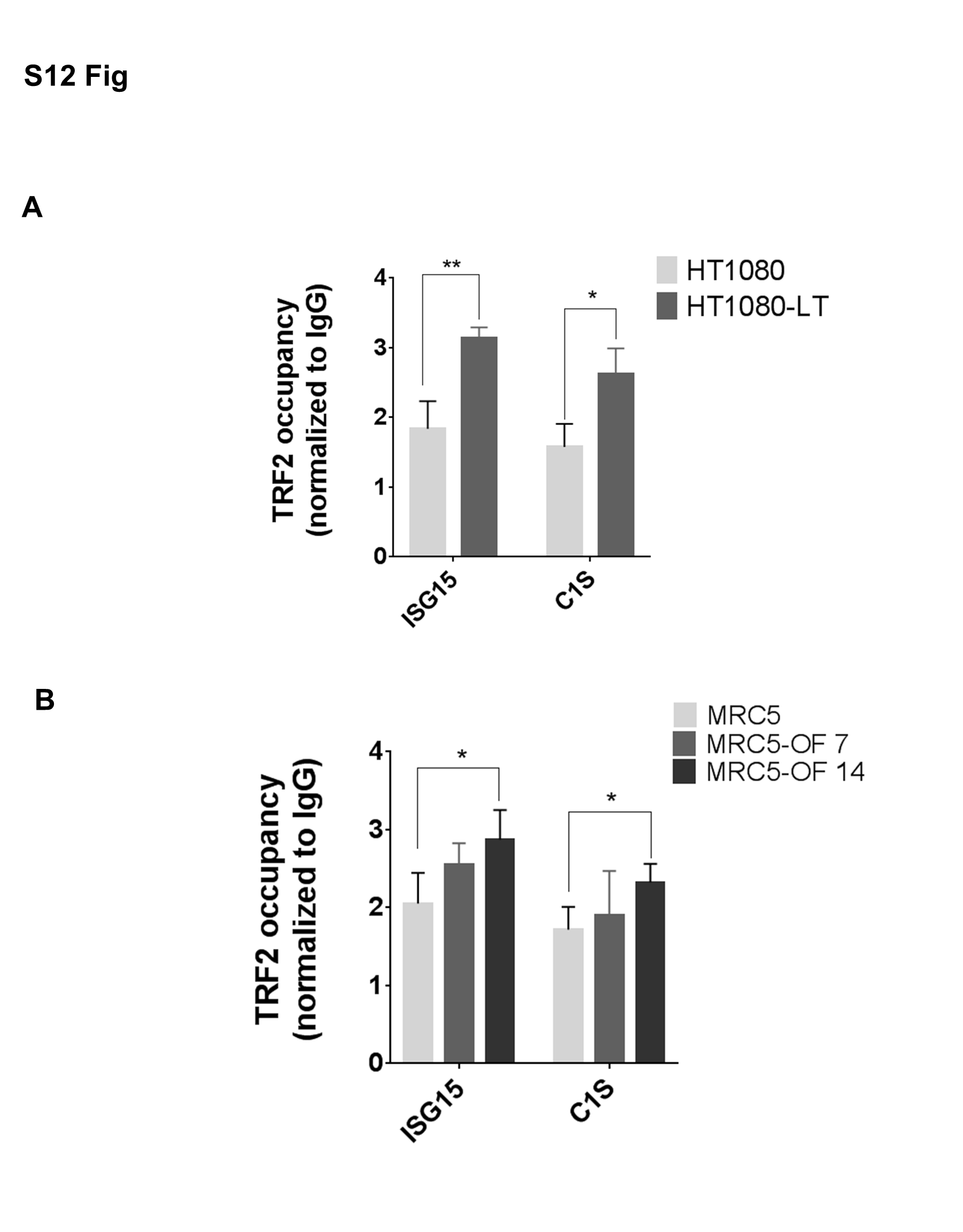

Supplement: S12 Fig — TRF2 occupancy at gene promoter sites reported for sub telomeric looping was checked HT1080 cells and HT1080-LT cells (A) and MRC5 OF cells (B). Error bars indicate ± SD from three independent experiments. significance was tested by paired T-test -* <0.05; **<0.01. (TIF) [file pgen.1007782.s012.TIF]

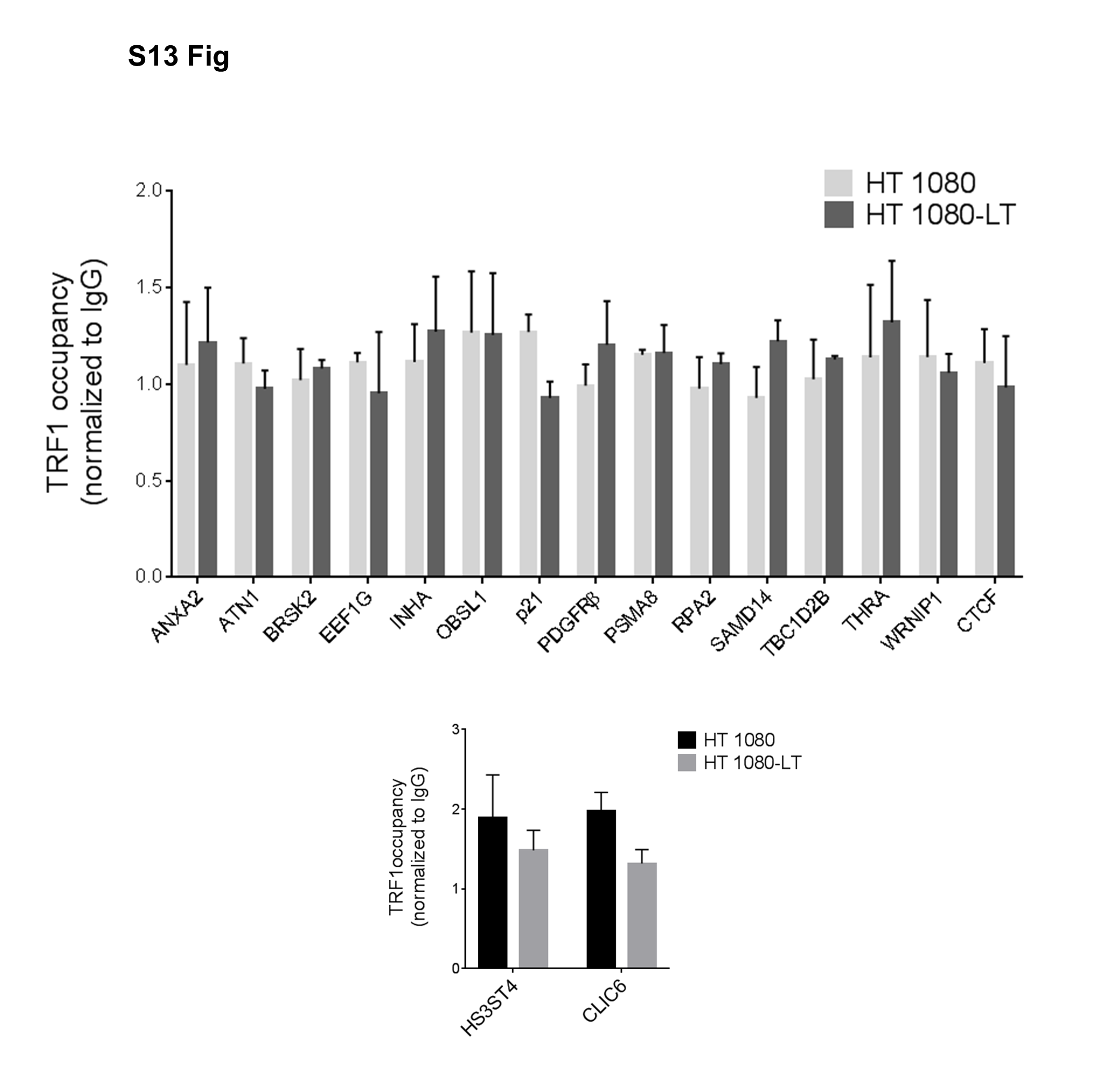

Supplement: S13 Fig — TRF1 occupancy on gene promoters in HT1080 and HT1080-LT cells (top panel). Internal telomeric repeats within HS3ST4 and CLIC6 genes reported to bind TRF1 were used as positive control for TRF1 ChIP (bottom panel). Error bars indicate ± SD from three independent experiments. (TIF) [file pgen.1007782.s013.TIF]

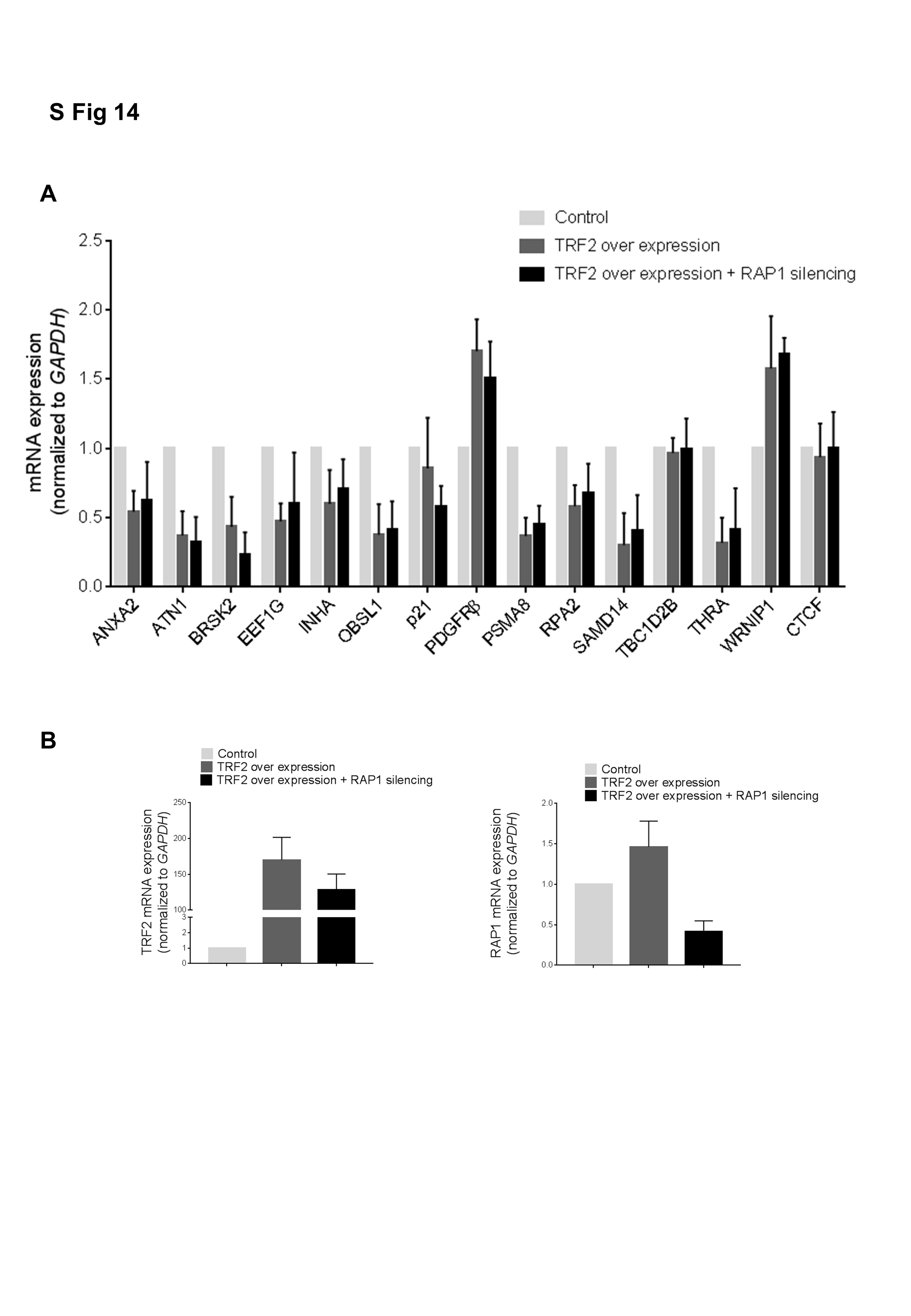

Supplement: S14 Fig — A. Effect of RAP1 silencing on TRF2-mediated gene expression. B. Confirmation of TRF2 over expression and RAP1 knockdown by qRT PCR Error bars indicate ± SD from two independent experiments. (TIF) [file pgen.1007782.s014.TIF]

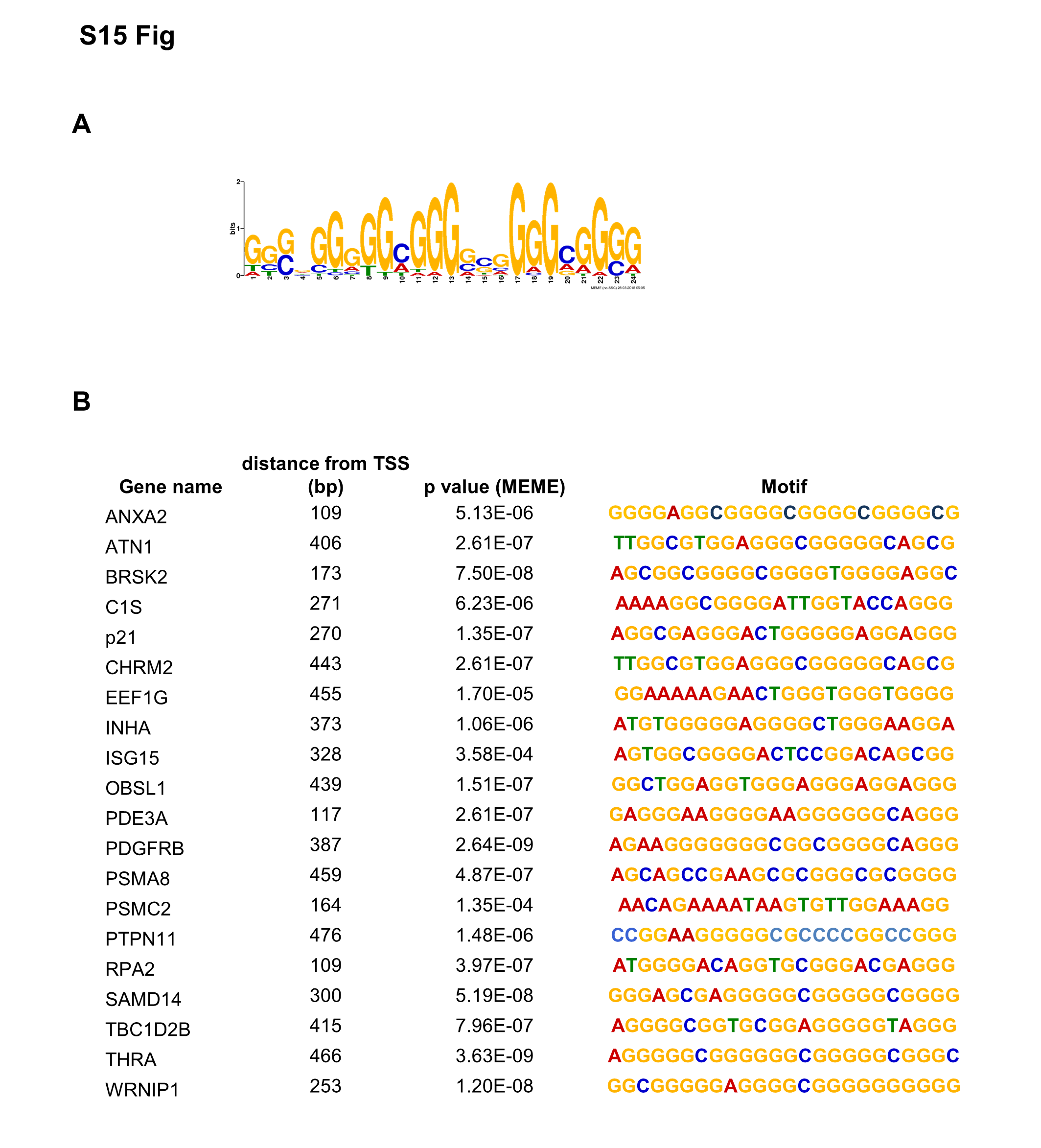

Supplement: S15 Fig — A. Consensus G-rich motif detected by MEME application for motif discovery within gene promoters validated for TRF2 binding B Individual motif sequences, distance of motif from TSS and significance of motif detection as obtained from MEME for gene promoters with differential promoter TRF2 occupancy in cells with short vs long telomeres. (detected motif nearest to the TSS have been shown). (TIF) [file pgen.1007782.s015.tif]

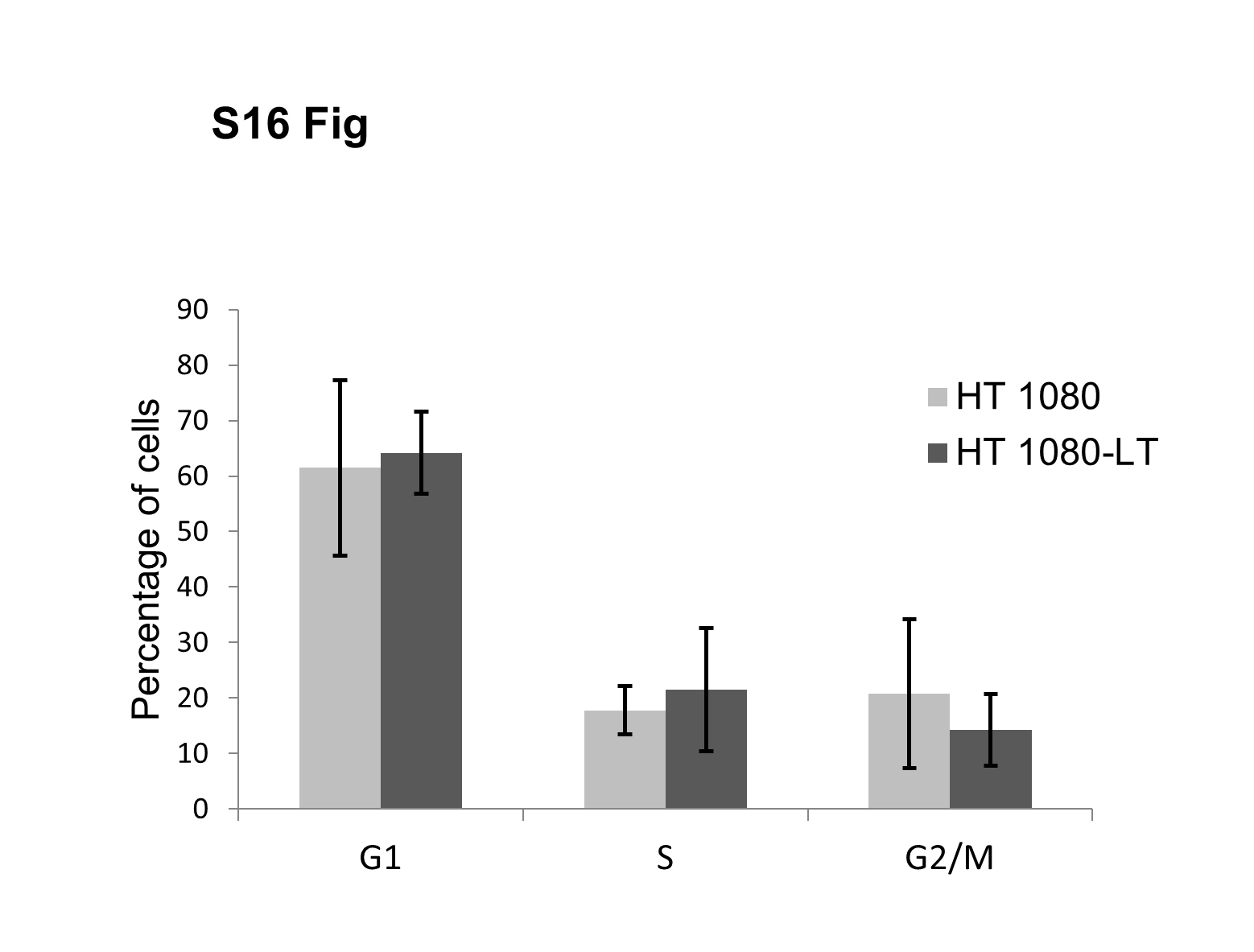

Supplement: S16 Fig — (TIF) [file pgen.1007782.s016.TIF]

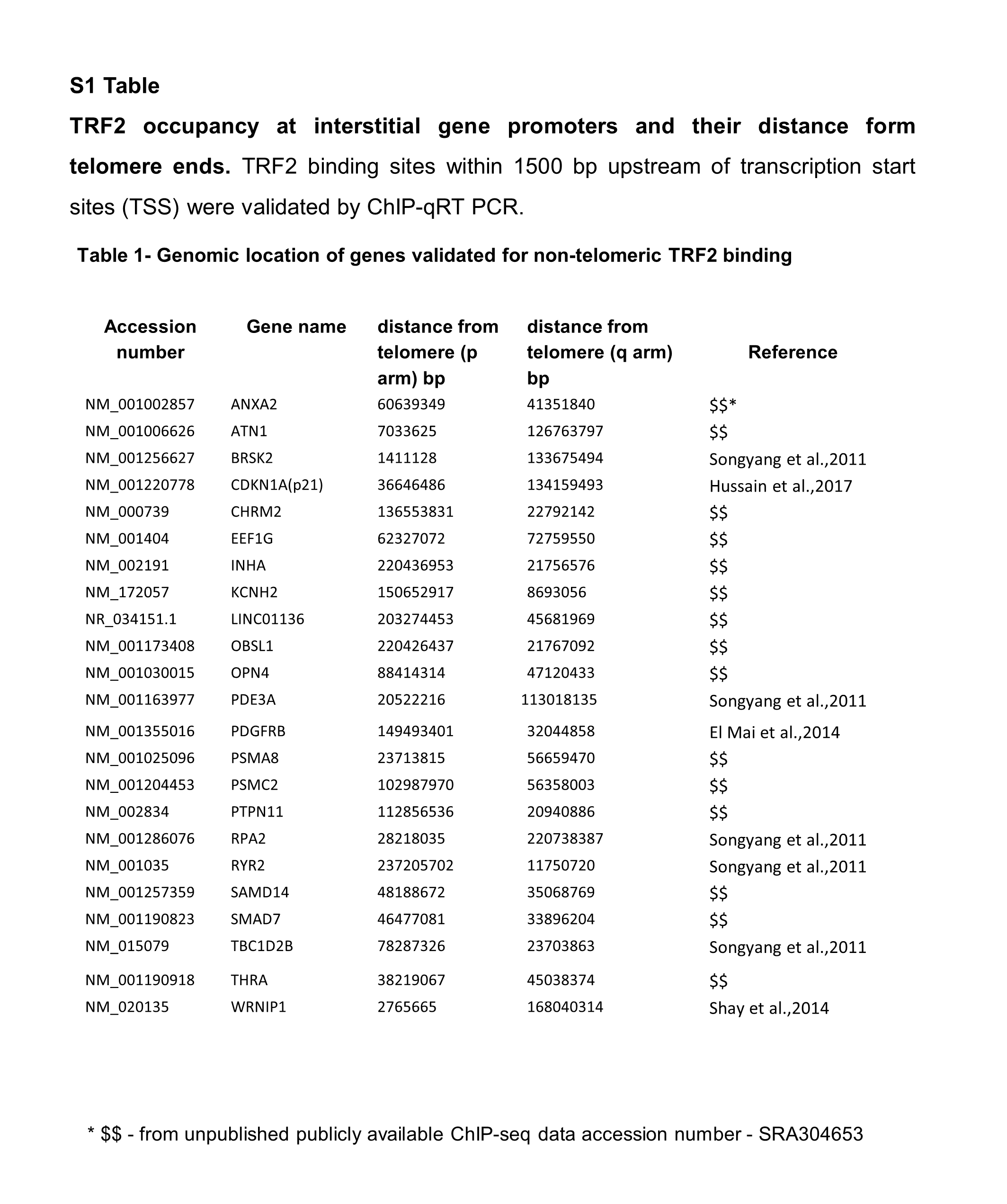

Supplement: S1 Table — TRF2 binding sites within 1500 bp upstream of transcription start sites (TSS) were validated by ChIP-qRT PCR. (TIF) [file pgen.1007782.s017.tif]

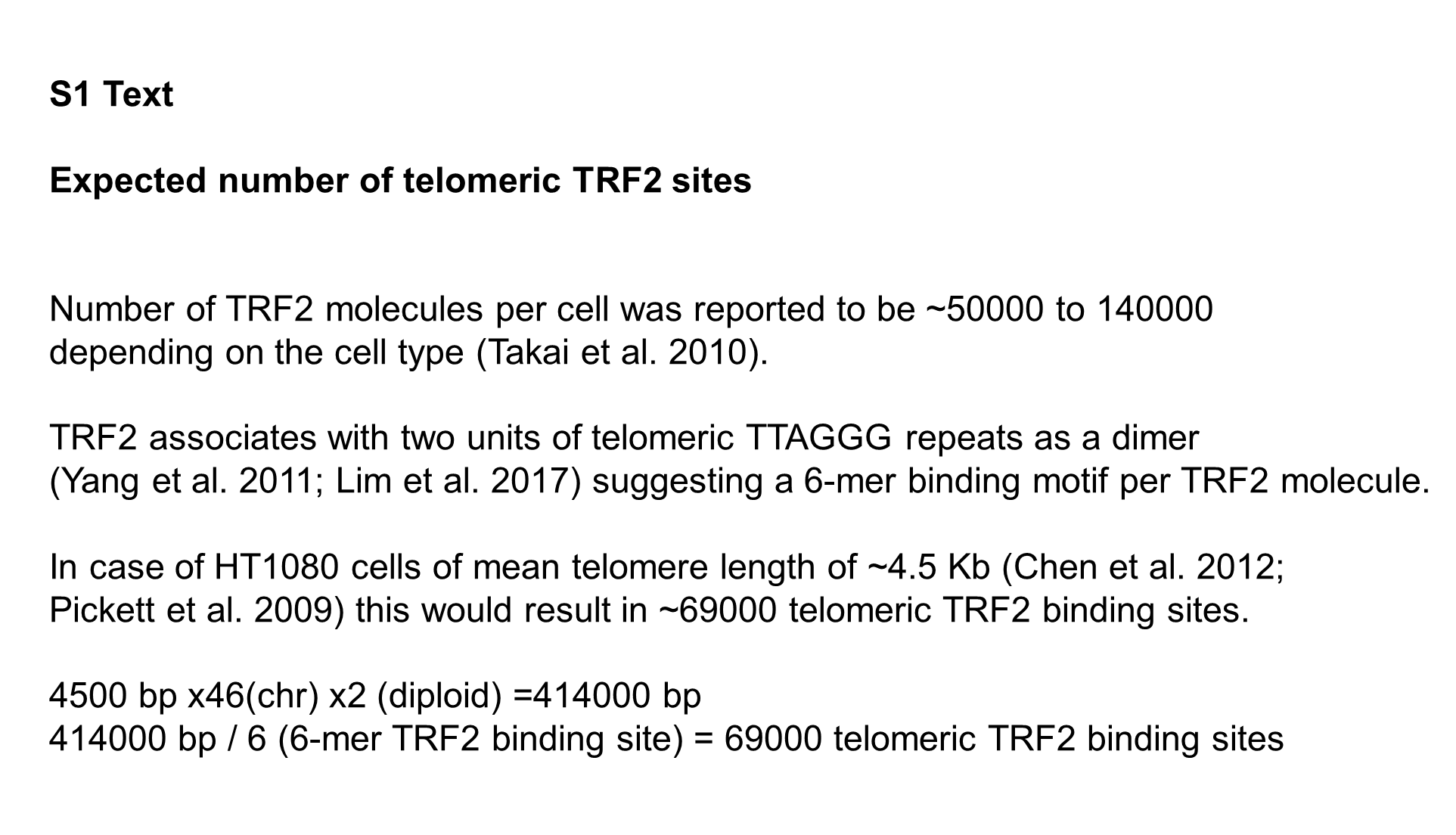

Supplement: S1 Text — Number of TRF2 molecules per cell was noted to be around 50000 to 140000 depending on the cell type (Takai et al. 2010). TRF2 associates with two units of telomeric TTAGGG repeats as a dimer (Yang et al. 2011; Lim et al. 2017) suggesting a 6-mer binding motif per TRF2 molecule. In case of HT1080 cells of mean telomere length of ~4.5 Kb (Chen et al. 2012; Pickett et al. 2009) this would result in ~69000 telomeric TRF2 binding sites. 4500 bp x46(chr) x2 (diploid) = 414000 bp 414000 bp / 6 (6-mer TRF2 binding site) = 69000 telomeric TRF2 binding sites. (TIF) [file pgen.1007782.s018.tif]
